# Physicochemical Characterization of the Pristine E171 Food Additive by Standardized and Validated Methods

**DOI:** 10.3390/nano10030592

**Published:** 2020-03-24

**Authors:** Eveline Verleysen, Nadia Waegeneers, Frédéric Brassinne, Sandra De Vos, Isaac Ojea Jimenez, Stella Mathioudaki, Jan Mast

**Affiliations:** 1 Trace elements and nanomaterials, Sciensano, Groeselenbergstraat 99, 1180 Uccle, Belgium; Frederic.Brassinne@sciensano.be (F.B.); Sandra.DeVos@sciensano.be (S.D.V.); isaac.ojea.jimenez@gmail.com (I.O.J.); stella.mathioudaki@sciensano.be (S.M.); jan.mast@sciensano.be (J.M.); 2 Trace elements and nanomaterials, Sciensano, Leuvensesteenweg 17, 3080 Tervuren, Belgium; nadia.waegeneers@sciensano.be

**Keywords:** E171, titanium dioxide, food additive, transmission electron microscopy, TEM, single-particle inductively coupled plasma mass spectrometry, spICP-MS, method validation, sample preparation, particle size distribution

## Abstract

E171 (titanium dioxide) is a food additive that has been authorized for use as a food colorant in the European Union. The application of E171 in food has become an issue of debate, since there are indications that it may alter the intestinal barrier. This work applied standardized and validated methodologies to characterize representative samples of 15 pristine E171 materials based on transmission electron microscopy (TEM) and single-particle inductively coupled plasma mass spectrometry (spICP-MS). The evaluation of selected sample preparation protocols allowed identifying and optimizing the critical factors that determine the measurement of the particle size distribution by TEM. By combining optimized sample preparation with method validation, a significant variation in the particle size and shape distributions, the crystallographic structure (rutile versus anatase), and the physicochemical form (pearlescent pigments versus anatase and rutile E171) was demonstrated among the representative samples. These results are important for risk assessment of the E171 food additive and can contribute to the implementation of the European Food Safety Authority (EFSA) guidance on risk assessment of the application of nanoscience and nanotechnologies in the food and feed chain.

## 1. Introduction

E171 (titanium dioxide) is a food additive that has been authorized for use as a food colorant in the European Union (EU) [1]. It is a white to slightly colored powder that is insoluble in water and organic solvents [2]. In food, both anatase and rutile titanium dioxide are applied [2]. Certain rutile grades of titanium dioxide are produced using potassium aluminum silicate (also known as mica) as a template to form a basic platelet structure [2] and are generally referred to as pearlescent pigments [3]. The application of E171 in food was subjected to a (re-)evaluation by the European Food Safety Authority (EFSA) in 2016 [4] and was re-approved for use in food. It is commonly applied in confectionery (including candies, chewing gum, glazings) but was demonstrated also in pastries, low-fat dairy products, and sauces [5,6,7,8,9]. 

Many consumers are exposed to food containing E171 on a daily basis [4,6,10,11,12,13]. Among the different routes of exposure, the oral uptake route remains the least documented [10]. As titanium dioxide has a low absorption rate, it is mostly excreted in the feces, suggesting that it does not present any toxicity concern [14,15,16]. However, the application of E171 in food has become an issue of debate within the European Union, since there are indications that it may alter the intestinal barrier [16,17,18,19,20,21,22,23,24,25,26,27,28,29,30,31,32,33,34,35,36,37,38,39,40,41,42,43,44,45,46,47]. In this respect, EFSA evaluated in 2018 the outcome of four studies [34,36,44,46] and concluded that there was no need for re-opening the EFSA opinion of 2016. 

More recently, Talamini et al. showed that the repeated administration of E171 to mice resulted in TiO_2_ deposition in the gastrointestinal tract and the liver, and it is associated with molecular and cellular alterations in the inflammatory response [48]. In addition, the French Agency for Food, Environmental, and Occupational Health and Safety (ANSES) proposed that titanium dioxide should be considered as being potentially carcinogenic to humans when inhaled, but there was no carcinogenic concern for oral and dermal exposure [49,50,51]. These studies urged relevant governmental agencies to reassess the safety of titanium dioxide as a food additive. Jovanović et al. proposed establishing an acceptable maximum daily intake as a precautionary measure [47]. 

Since E171 is a particulate material containing a fraction of nanoparticles [6,14,22,52], and toxicology studies mainly focus on the systemic absorption of this nanofraction after ingestion [48], EFSA noted a need for more data, and particularly for information related to the particle size distribution of E171, as well as an indication of the percentage (in number and by mass) of the particles in the nanoscale together with information on the analytical methods and techniques used for the detection and quantification of the nanofraction [4]. 

From this perspective, EFSA published a scientific opinion on the proposed amendment of the EU specifications for titanium dioxide (E171) with respect to the inclusion of additional parameters related to its particle size distribution [53]. This scientific opinion was based on particle size analyses provided by interested business operators, reporting median constituent particle sizes for five brands of anatase and one rutile sample ranging from 104 to 166 nm and a percentage of particles <100 nm ranging from 5.4% to 45.6%. Based on these results, the panel proposed to insert a specification of more than 100 nm for median minimal external dimension in the current EU specifications, which is equivalent to less than 50% of the number of constituent particles with a minimal external dimension below 100 nm. The panel also reiterated the need for further research to decrease the level of uncertainty of the size measurement.

In this context, Geiss et al. show how even in relatively simple matrices, sample preparation can have an impact on the measurement of the particle size distribution and on the interpretation of whether an extracted material is considered to be a nanomaterial according to the European Commission (EC)-recommended definition [54]. They stress the need for validated and harmonized sample preparation protocols prior to the particle size characterization [55]. 

In addition to the EFSA opinion, several studies characterized commercially available pristine E171 materials and food products containing E171 [6,7,9,42,43,52,56,57,58]. In general, mean particle sizes between 106 and 145 nm and size distributions ranging from 30 nm to 400 nm were reported for electron microscopy (EM) analyses [6,9,42,43,56,57,58]. One study showed a size distribution ranging from 30 to 600 nm for scanning electron microscopy (SEM) and 20 to 400 nm for asymmetric flow field-flow fractionation coupled with inductively coupled plasma mass spectrometry (AF4-ICP-MS) [7]. In these studies, the fraction of nanoparticles (<100 nm) ranged from 10% to 54% [6,7,9,42,43,56,57]. Geiss et al. were able to characterize E171 contained in finished and semi-finished confectionery products, along with exactly those pristine E171 samples used in each of the products, allowing a direct comparison of the particle size distribution in the pristine and extracted materials. Their study included the characterization of one pearlescent pigment and five different types of E171. For the pearlescent pigment, they found µm-sized mica plates coated with 15–25 nm particles. For the five different types of E171, they obtained fractions of nanoparticles below 100 nm ranging from 23.6% to 66.0% and a D50 between 80.0 and 139.5 nm [55]. 

Even though a reasonable amount of studies have reported E171 characterization results, method variation and bias are often not considered: methods are not standardized, and results systematically lack measurement uncertainties obtained through validation studies.

This work applies standardized and validated methodologies to characterize representative samples of 15 pristine E171 materials, including the E171 materials discussed in the EFSA opinion [53], based on transmission electron microscopy (TEM) and single-particle inductively coupled plasma mass spectrometry (spICP-MS). It assesses the impact of the type of E171 material, the sample preparation, and measurement methods with uncertainties.

The TEM specimen (grid) preparation, imaging, and image analysis procedures were previously validated intra laboratory [59], while validation performance characteristics for a standardized spICP-MS methodology are given in this publication. 

## 2. Materials and Methods

### 2.1. Selection of Materials

Nine pristine E171 materials were purchased from webshops that specialized in bakery and confectionery products from several countries within the European Union (Table 1). According to the list of ingredients on the labels, these materials only contained the E171 food additive. Six pristine E171 materials, as discussed in the EFSA opinion [53], were obtained from the business operators. In this study, E171 additives obtained from webshops are labeled as E171-01, 02, …, 09 and E171 additives of business operators are labeled as E171-A, B, …, F. All materials were obtained as powders.

### 2.2. Transmission Electron Microscopy (TEM) Analysis of E171 Materials

#### 2.2.1. Sample Preparation

Zeta potential curves were constructed by dispersing 25 mg of powder in 10 mL of 0.01 M HNO_3_ using a Vibracell™ 75,041 ultrasonifier (Fisher Bioblock Scientific, Aalst, Belgium) equipped with a 13 mm probe (CV33) following the ENPRA dispersion protocol for NANoREG [60] and subsequently measuring the zeta potential by Dynamic Light Scattering (DLS) (Zetasizer Nano ZS, Malvern Panalytical, Malvern, UK) in the pH range from 1 to 11 by increasing the pH stepwise. 

Six sample preparation protocols, labeled from P1 to P6 were evaluated. In these protocols, probe sonication was done using the Vibracell™ 75,041 ultrasonifier (Fisher Bioblock Scientific, Aalst, Belgium) equipped with a 13 mm probe (CV33), and centrifugation was done using a micro-centrifuge (Carl ROTH, Karlsruhe, Germany) at a speed of 6000 rpm (corresponding to a G-force of approximately 2000). The protocols are summarized in Table 2 and include: 

(P1) 25 mg of pristine E171 was brought in 10 mL of NaOH 0.1 mM solution (pH 10) in a 20 mL glass vial and dispersed by probe sonication at 40% amplitude, until 35 kJ of energy was delivered. Samples were cooled in ice water during sonication.

(P2, P3) 88 mg of pristine E171 was brought in 35 mL of ultrapure water in a 50 mL polypropylene tube. After 30 s of vortex stirring, 500 µL of the dispersion was brought into a 1.5 mL Eppendorf vial and centrifuged at 6000 rpm (approximately 2000 g) for 30 min (P2) or 2 h (P3). The supernatant was removed and the pellet was re-suspended in 500 µL of ultrapure water. 

(P4) 88 mg of pristine E171 was brought in 35 mL of ultrapure water in a 50 mL polypropylene tube. After 30 s of vortex stirring, 10 mL was transferred in a 20 mL glass vial and dispersed using probe sonication at 20% amplitude until 10 kJ of energy was delivered. Samples were cooled in ice water during sonication. 

(P5, P6) 88 mg of pristine E171 was brought in 35 mL of ultrapure water in a 50 mL polypropylene disposable tube. After 30 s of vortex stirring, 10 mL was transferred in a 20 mL glass vial and dispersed using probe sonication at 20% amplitude until 10 kJ of energy was delivered. Samples were cooled in ice water during sonication. After sonication, 500 µL of the dispersion was brought into an 1.5 mL Eppendorf vial and centrifuged at 6000 rpm (approximately 2000 g) for 30 min (P5) or 2 h (P6). The supernatant was removed, and the pellet was re-suspended in 500 µL of ultrapure water. 

For centrifugation-based protocols (P2, P3, P5, and P6), centrifugation times were calculated based on Stoke’s law. For the applied Eppendorf vials and a volume of 500 µL, the maximal distance that the particles need to descend is 1.75 cm. Stoke’s law predicts that 30’ of centrifugation (P2 and P5) allows 20 nm, 40 nm, and 60 nm anatase TiO_2_ particles to descend 0.2 cm, 1.0 cm, and 2.2 cm, respectively, while 2 h of centrifugation (P3 and P6) allows 20 nm, 40 nm, and 60 nm anatase TiO_2_ particles to descend 1.0 cm, 3.9 cm, and 8.8 cm, respectively.

To examine the sample preparation-induced effects on the measurement of particle size distributions, all six protocols (P1–P6) were evaluated on materials E171-01 and E171-06. 

Subsequently, the most optimal protocols (P1 and P6) were applied on all E171 materials.

#### 2.2.2. TEM Specimen (Grid) Preparation

TEM specimens (grids) were prepared as described by Mast et al. [61] using Alcian blue treated positively charged pioloform- and carbon-coated 400-mesh copper grids (Agar Scientific, Stansted, Essex, UK), by drop deposition based on the SOP “Preparation of EM-grids containing a representative sample of a dispersed nanomaterial” [62].

#### 2.2.3. Descriptive TEM

For each material, a set of calibrated selected and representative images showing an even distribution of particles on the TEM specimen was recorded by a 120 kV Tecnai G2 Spirit TEM with BioTwin lens configuration (Thermo Fisher Scientific, Eindhoven, The Netherlands), which was equipped with a 4 × 4 k Eagle charge-coupled device (CCD) camera (Thermo Fisher Scientific) while using the TEM imaging and analysis (TIA) software (Version 3.2, Thermo Fisher Scientific). All samples were initially screened at multiple magnifications, and a detailed description was prepared, allowing to assess the quality of the TEM specimen preparation, following the guidelines of Mast et al. [63].

#### 2.2.4. Electron Diffraction

The crystallographic structure of the E171 materials purchased in webshops (E171-01-…-09) was determined by electron diffraction. Selected area electron diffraction (SAED) patterns of regions containing many particles were recorded, indexed, and compared to a database [64]. The camera length was determined based on the diffraction pattern of colloidal gold nanoparticles. The crystallographic structure of materials E171A-F was specified in the EFSA opinion [53]: materials E171-A-E were reported to be anatase, and material E171-F was reported to be rutile.

#### 2.2.5. High-Angle Annular Dark Field (HAADF)–Scanning Transmission Electron Microscopy (STEM) and Energy Dispersive X-ray Spectroscopy (EDX)

High-angle annular dark field (HAADF)–scanning transmission electron microscopy (STEM) imaging and energy-dispersive X-ray spectroscopy (EDX) analyses were performed using a 200 kV Talos F200S G2 TEM equipped with an HAADF detector and a Super-X EDS detector consisting of 2 windowless silicon drift detectors (SDD), using Velox software (Version 2.10, Thermo Fisher Scientific, Eindhoven, The Netherlands). STEM images were recorded with a scan size of 1024 × 1024 pixels, a dwell time of 20 µs, a probe convergence angle of 7.5 mrad, and a camera length of 160 mm. EDX spectra were recorded during 10 s with a 20 keV energy range, 5 eV dispersion, and optimized shaping time. Spectral imaging was performed using a live time of 7 min and 36 s in which 20 frames were recorded. 

#### 2.2.6. Quantitative TEM: Imaging, Image Analysis, and Data Analysis

Quantitative TEM analysis was performed using the Tecnai microscope, following the standard operating procedure (SOP) on TEM imaging: “Transmission electron microscopic imaging of nanomaterials”, which aims to record a set of calibrated images that representatively show the (nano)material on the TEM specimen [62]. The images were randomly and systematically recorded at positions pre-defined by the microscope stage and evenly distributed over the entire grid area to avoid subjectivity in the selection of particles by the analyst. 

To determine unbiased, number-based size distributions by quantitative TEM for all samples, the magnification and the associated pixel size (which corresponds to the lower limit of detection (LLOD)) were determined based on the criterion of Merkus [65]. The lower limit of quantification (LLOQ) was defined as 10 times the LLOD. The corresponding upper limit of quantification (ULOQ) was limited to 1/10^th^ of the image size (which corresponds with the upper limit of detection (ULOD)), supporting on ISO 13322-1 [66]. The magnifications and corresponding LLOQ and ULOQ selected for each E171 material are given in Table 1. 

The size and shape properties of the constituent particles of the E171 materials were measured based on the properties of their 2D projections using the ParticleSizer software following the SOP “Measurement of the minimal external dimension of the constituent particles of particulate materials from TEM images by the NanoDefine ParticleSizer software” [67,68]. The constituent particles were analyzed by noise and background suppression combined with either irregular watershed segmentation or ellipse fitting, depending on the E171 material (Table 1). For each material, the distributions of the (maximum) Feret diameter (Fmax), the minimum Feret diameter (Fmin), and the aspect ratio (AR) were determined. For the materials analyzed using ellipse fitting, the primary (major) axis of the fitted ellipse was used as an estimate of Fmax, and the secondary (minor) axis was used as an estimate of Fmin.

Sample preparation effects were evaluated on material E171-06 by measuring the agglomerate size alongside the constituent particle size using the single-particle mode of the ParticleSizer software. Particle detection was evaluated by the visual inspection of annotated images, and wrongly detected particles were removed manually from the datasets.

The raw data resulting from the image analysis were processed using an in-house python script for the calculation of descriptive statistics and plotting histograms, following ISO 9276-1 and ISO 9276-3 guidelines for the representation of results of particle size analysis [69,70]. For all materials, histograms and kernel density estimates of the (normalized) number-based distributions of the Fmin, Fmax, and AR parameters were determined. The modes of the distributions were obtained from the kernel density estimates.

#### 2.2.7. Measurement Uncertainties

Measurement uncertainties on the medians of the Fmin distributions of the E171 materials were estimated based on the validation study of Verleysen et al. [59] for representative test materials NM-100 (TiO_2_ materials with particles in the order of 100 nm) and NM-103 (TiO_2_ materials with particles in the order of 20 nm). The expanded measurement uncertainties (Ucx, k = 2) for median Fmin measurements are reported to be 8.5% and 9.2% for NM-100 and NM-103, respectively [59]. 

From the same datasets obtained in the study of Verleysen et al., the measurement uncertainties on the medians of the Fmax and AR distributions of NM-100 and NM-103 were determined by following exactly the same procedure as described by Verleysen et al. for Fmin (data not shown). For NM-100 and NM-103, the expanded measurement uncertainties are 9.1% and 10.4% for median Fmax and 3.7% and 4.2% for AR, respectively. 

### 2.3. Single-Particle Inductively Coupled Plasma Mass Spectrometry (spICP-MS) Analysis of E171 Materials

#### 2.3.1. Sample Preparation

All E171 materials were dispersed by bringing an accurately weighed subsample of 3.5 mg into a 20 mL liquid scintillation vial (Wheaton, Millville, NJ, USA). Subsequently, 10 mL of ultrapure water (UPW) was added, and the sample was vortexed for about 10 s. The final E171 concentration in the dispersions was 0.37 ± 0.05 mg/mL. The dispersions were left to rest on ice for 5 min, after which they were sonicated for 18.5 min with the Vibracell™ 75,041 ultrasonifier (Fisher Bioblock Scientific, Aalst, Belgium) equipped with a 13 mm probe (CV33) at 40% amplitude, which resulted in an applied energy of 32 ± 1 kJ (mean ± σ). Three independent replicates were prepared this way. Each dispersion was diluted in polypropylene vials to two different levels with a 4% (v:v) HNO_3_ solution. The two appropriate dilutions were determined after a range-finding test, during which different dilutions of the dispersion were measured. The two dilutions resulted in a proportionally changing number of detected particles, a constant particle size, and 200–2200 detected particles.

#### 2.3.2. Instrumentation and Analysis

An ICP-MS/MS (Agilent 8800, Agilent Technologies, Santa Clara, CA, USA) was used for data acquisition in time-resolved analysis mode. Ammonia (NH_3_) was thereby used as the reaction gas. Titanium was measured after a mass shift from m/z 48 to m/z 150. In order to increase the sensitivity of the spICP-MS analyses, instrument tuning was optimized for analyses at m/z 150 by adjusting the sample depth and carrier gas flow rate, amongst other factors. The instrument parameters and operational conditions are given in Table 3. 

The transport efficiency was determined according to the particle frequency method [71] by means of 30 nm gold nanoparticles from nanoComposix at a concentration of 12.5 ng/L under the same instrumental conditions as the samples. Mass calibration was performed by measuring ionic Ti standard solutions prepared in 4% HNO_3_. Following the analysis of each sample, UPW was measured to monitor the potential carryover from the previous sample. 

The single-particle calculation spreadsheet described by [72] was used to calculate the particle size distributions and particle number or mass concentrations. The particle diameter, referred to as equivalent spherical diameter (ESD), was obtained from the particle mass assuming a spherical geometry. A detailed description of the single-particle calculation spreadsheet, including calculation equations, can be found in [72]. To discriminate between particles and ionic Ti or incomplete particle events (ion plumes detected over two consecutive dwell times), an iterative algorithm based on µ + 5σ was applied on the data as described by [73] and [74] and verified visually to ensure the absence of an extraordinary high peak at the lower-size side of the size distribution.

#### 2.3.3. Measurement Uncertainties

Performance characteristics of the method are summarized in Table 4. The size detection limit was determined in UPW as mean + 3σ_BG_. However, as the background and the standard deviation on the background signal were extremely low, four counts (1334 cps) were set as the minimal intensity of the smallest detectable nanoparticle. The size quantification limit is sample dependent and determined for each individual replicate. Precision parameters were determined by analyzing three independent replicates of the representative test material NM-100 (JRC, Ispra, Italy) on each of five different days and assessed via one-way analysis of variance. As a proxy for trueness estimation (“apparent trueness”), the median ESD determined by spICP-MS was compared to the median Fmin determined by TEM for NM-100 (i.e., 100 nm; [59]). The recovery of particle mass was determined as a percentage of the theoretical concentration, which is 1 g/g. No attempt was made to estimate the recovery for the particle number concentration. Repeatability standard deviation (s_r_), between-day standard deviation (s_d_), and the uncertainty on the bias (u_Δ_) were estimated as explained in [75]. To determine the measurement uncertainty under routine measurement conditions (three replicates analyzed on a single day), the repeatability and between-day variations were divided by respectively the number of replicates and the number of measurement days. Hence, the combined measurement uncertainty (u_c_) was calculated as:

(1) $$u_{c}=\sqrt{\frac{s_{r}^{2}}{3}+\frac{s_{d}^{2}}{1}+u_{\Delta }^{2}}.$$

The expanded measurement uncertainty (Ucx, k = 2) was obtained by multiplying the combined measurement uncertainty by 2.

Relative uncertainties were calculated by dividing the calculated uncertainties by the mean ESD, number, or mass concentration, respectively.

## 3. Results

### 3.1. Zeta Potential Measurements

**Figure 1:**
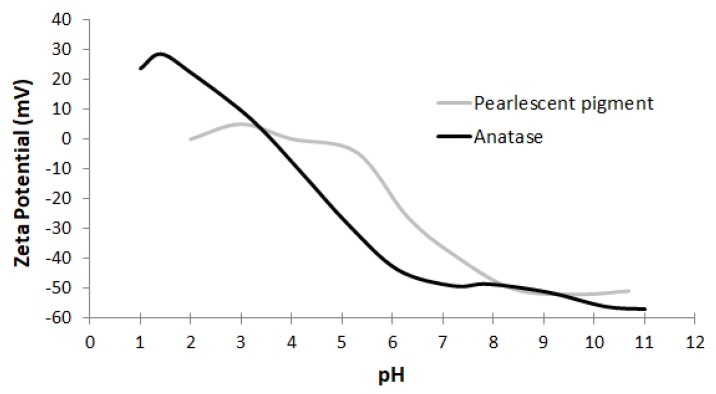
Zeta potential curves representative for the two groups of E171 materials, observed among the nine E171 materials obtained from webshops: pearlescent pigments and anatase TiO_2_.

The zeta potential curves of the materials purchased in webshops indicate two groups of E171 materials (Figure 1). The first group has isoelectric points (IEPs) between pH 3 and pH 4 and the particles have strong negative charges from pH 6 to pH 11, suggesting that these materials are stable in dispersion within this pH range. The zeta potential curves of the second group are shifted towards higher pH values and particles have strong negative charges from about pH 8 to pH 11, suggesting that these materials are stable in dispersion at a higher pH than the first group. At the pH range where particles have negative charges, they interact strongly to the positively charged, Alcian blue-coated EM grids. 

### 3.2. Descriptive TEM Analysis and Electron Diffraction

**Figure 2:**
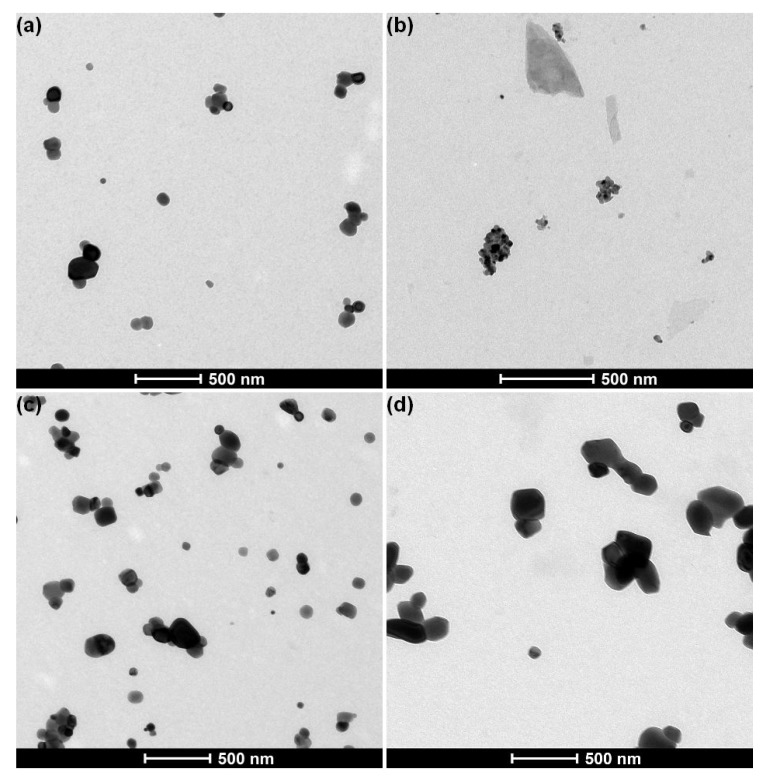
Representative transmission electron microscopy (TEM) images of E171 materials with (**a**) anatase TiO_2_ obtained from webshops (image of material E171-06, prepared using protocol P6), (**b**) pearlescent pigment Type I, obtained from webshops (image of material E171-01, prepared using protocol P1), (**c**) anatase TiO_2_ from business operators (image of material E171-B, prepared using protocol P6), and (**d**) rutile TiO_2_ (image of material E171-F, prepared using protocol P6) from business operators.

In line with the zeta potential measurements, two groups were observed by descriptive TEM analysis of the materials purchased in webshops (Figure 2).

Six out of nine materials (E171-02, E171-03, E171-04, E171-06, E171-07, and E171-09) were stable in dispersion when sample preparation protocols P1 and P6 were applied and resulted in a homogeneous distribution of particles on the EM grid. The samples contained near-spherical constituent particles with a diameter of approximately 100 nm, which were often agglomerated (Figure 2a). No impurities were observed. Electron diffraction analysis showed that the diffraction patterns of these particles match with anatase titanium dioxide. 

Three materials (E171-01, E171-05, and E171-08) were stable in dispersion when sample preparation protocol P1 was applied, but they precipitated when sample preparation protocol P6 was applied. The samples prepared using protocol P1 contained aggregated, near spherical constituent particles measuring 20 to 30 nm (Figure 2b). In addition, the samples contained other structures with a smooth, non-particulate surface. The particles often formed a layer on large flakes supporting the particles. Electron diffraction analysis showed that the diffraction patterns of these particles match with rutile titanium dioxide. Such a description suggests that these materials are pearlescent pigments, according to the specifications of the Joint Food and Agriculture Organization of the United Nations (FAO) / World Health Organization (WHO) Expert Committee on Food Additives (JECFA) [3].

All materials obtained from the business operators were stable in dispersion when sample preparation protocols P1 and P6 were applied. The five anatase materials (E171A–E) contained near spherical constituent particles with a diameter measuring approximately 100 nm, which were often agglomerated (Figure 2c). The rutile material (E171-F) contained near spherical constituent particles measuring 100 to 500 nm, which were often agglomerated (Figure 2d). No impurities were observed in the materials.

### 3.3. HAADF-STEM and EDX Analysis

**Figure 3:**
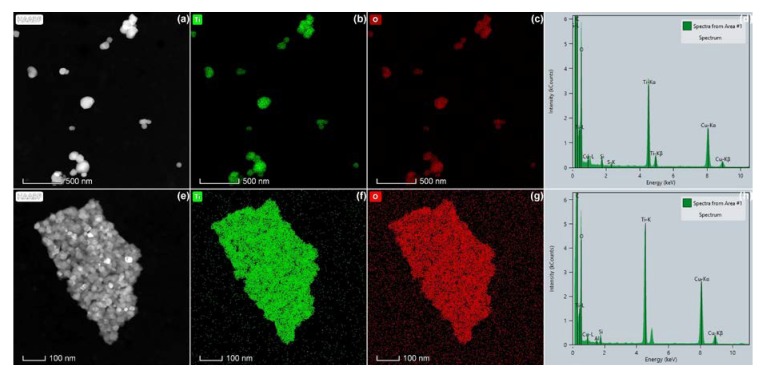
High-angle annular dark field (HAADF)–scanning transmission electron microscopy (STEM) images (**a**,**e**), spectral images of Ti (**b**,**f**) and O (**c**,**g**) obtained by energy-dispersive X-ray spectroscopy (EDX), and EDX spectra (**d**,**h**) of E171 materials purchased at webshops: (a–d) anatase TiO_2_ (E171-06) and (e–h) pearlescent pigment (E171-05).

EDX analyses showed that all materials contain particles consisting of the elements titanium and oxygen, confirming that they consist of titanium dioxide. This is illustrated in Figure 3 for the anatase TiO_2_ material E171-06 and for the pearlescent pigment E171-05. The copper and carbon signals in the spectra originate from the TEM specimen (grid) on which the particles were coated. In the anatase TiO_2_ materials, a small Si signal was often measured, which may originate from the EDX detector, since it is often measured in blanks as well [76]. 

In the pearlescent pigments, EDX analysis demonstrated structures containing K, Al, Si, and often small quantities of Fe. This elemental composition is in agreement with potassium aluminum silicate, also known as mica, which is often applied as a template for food-grade rutile TiO_2_ applied in E171 [2]. This further confirms that the materials E171-01, E171-05, and E171-08 are potassium aluminum silicate-based pearlescent pigments of Type I, according to the JECFA specifications [3].

**Figure 4:**
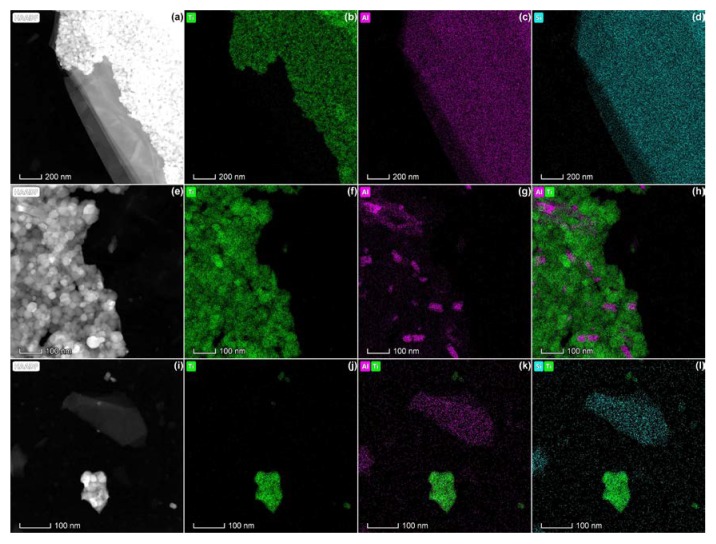
HAADF-STEM images (a,e,i) and corresponding spectral images of Ti (green), Al (pink), and Si (blue) obtained by EDX of pearlescent pigments, showing (**a**–**d**) aggregated TiO_2_ particles on top of mica, (**e**–**h**) a TiO_2_ aggregate containing small parts of mica, and (**i**–**l**) separate TiO_2_ aggregates and a mica flake.

Mica was observed in all pearlescent pigments, both as large flakes supporting the TiO_2_ particles (Figure 4a–d) and associated with TiO_2_ aggregates (Figure 4e–h). In addition, mica flakes without a coating with TiO_2_ particles were observed (Figure 4i–l). Possibly, the TiO_2_ coating detached during the sonication step in the sample preparation protocol applied for these materials (P1). The EDX spectra corresponding with these analyses are given as supplementary material (Figure S1).

### 3.4. Quantitative TEM Analysis

#### 3.4.1. Evaluation of Sample Preparation for Quantitative TEM Analysis

As an example for the anatase materials, E171-06 was prepared by the six sample preparation protocols (P1–P6) summarized in Table 2. Table 5 shows the medians of the Fmin, Fmax, and AR distributions obtained for constituent particles and agglomerates of material E171-06. The modes, 25^th^ percentile, and 75^th^ percentile are given as supplementary material (Table S1). The following observations were made:

1. Centrifugation without sonication (P2 and P3) resulted in an overestimation of the agglomerate and the constituent particle size of approximately 100 nm and 10 nm, respectively, compared to the sonication-based sample preparation protocols (P1, P4, P5, and P6).

2. The limited decrease in agglomerate size between 30’ (P2 and P5) and 2 h (P3 and P6) centrifugation-based protocols is explained by the increase of single particles on the EM grid. Single particles are smaller than agglomerates and consequently take more time to sediment. This minor decrease in agglomerate size did not significantly facilitate constituent particle size measurement. 

3. When samples were sonicated (P1, P4, P5, and P6), comparable constituent particle sizes were obtained with and without centrifugation, showing that the centrifugation step is not critical for the constituent particle size measurement of these pristine E171 materials. 

The calculated centrifugation time of 2 h is based on the size of the smallest particles (about 20–30 nm) present on the TEM specimen (grid), as confirmed by descriptive TEM analysis at multiple magnifications. It is not expected that a sub-fraction of even smaller particles would be present in the dispersions, given that they are stable at the selected pH, and as such at least some of these smaller particles would attach to the grid. Furthermore, since the E171 materials are agglomerated, it is expected that agglomerates of these smaller particles would be found on the grid after applying the 2 h centrifugation-based protocol, which was not the case. Geiss et al. performed a similar centrifugation-based protocol and confirmed the completeness of the particles’ sedimentation during the centrifugation step by analyzing the supernatant for the presence of particles [55]. 

As an example for the pearlescent pigments, material E171-01 was prepared by the six sample preparation protocols (P1–P6) summarized in Table 2. Only protocol P1 (Table 2) was suitable, since the material was strongly (negatively) charged at pH 10, and not at pH 7, resulting in a stable dispersion (Figure 1). Sonication was required to break up large aggregates, allowing accurate, automated particle size measurement. Protocol P1 was selected as the most optimal protocol for preparation of the three pearlescent pigments E171-01, E171-05 and E171-08.

#### 3.4.2. Evaluation of Image Analysis

For the anatase E171 materials and rutile material E171-F, noise and background suppression combined with ellipse fitting allowed a highly automated image analysis of the constituent particles. Examples of annotated images are given as supplementary material (Figure S2). In the case of single constituent particles or small agglomerates of constituent particles, ellipse fitting-based particle detection succeeded in precise and accurate particle detection and measurement. In agglomerates consisting of three or more constituent particles, ellipse fitting sometimes lead to the detection of duplets, triplets, etc., as singlets. Even though the agglomeration-determined overestimation of the constituent particle size was limited, it was proportional to the degree of agglomeration resulting from the selected sample preparation routine. 

For the pearlescent pigments, noise and background suppression combined with irregular watershed allowed automated image analysis of the constituent particles (Figure S2). The ParticleSizer software succeeded in distinguishing most of the rutile particles from the mica flakes. For small aggregates, irregular watershed succeeded in correct constituent particle detection and measurement. However, since these materials contained many aggregates containing a high number of constituent particles, incorrect segmentation occurred frequently, causing over- or underestimation of the median constituent particle size. 

#### 3.4.3. Quantitative TEM Results

The magnification selected for the quantitative TEM analysis was suitable because a Fmin smaller than the LLOQ was measured only for 0.3% of constituent particles, and no particles with a Fmin larger than the ULOQ were found (Table 1). In all samples, more than 300 (and for optimized protocols about 1000) particles were measured, which is sufficient to obtain an expanded measurement uncertainty (Ucx, k = 2) below 10%, as determined for representative test materials NM-100 and NM-103 by Verleysen et al. [59].

**Figure 5:**
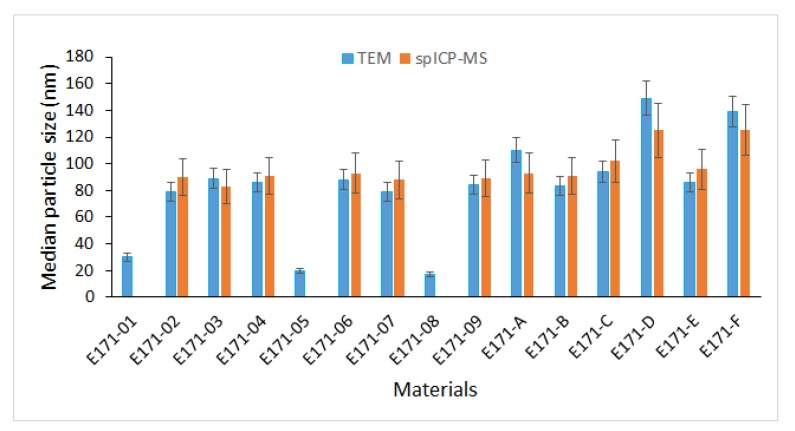
Medians of particle size distributions, expressed as Fmin and ESD for TEM and spICP-MS, respectively, of all 15 E171 materials. For TEM, medians of Fmin were obtained using P1 for pearlescent pigments (E171-01, E171-05, and E171-08) and P6 for the other materials. The error bars represent the expanded measurement uncertainties (Ucx, k = 2), which for TEM were obtained from RTMs NM-100 and NM-103, and for spICP-MS, as shown in Table 4.

Table 6 and Figure 5 summarize the quantitative TEM measurements for all 15 E171 materials using optimized sample preparation protocols (P1 for pearlescent pigments and P6 for the anatase E171 materials and rutile material E171-F) and image analysis settings. The corresponding number-based distributions are given as supplementary material (Figure S3). 

The difference in median particle size (Fmin and Fmax) between the pearlescent pigments and the other E171 materials is significant, considering the expanded measurement uncertainties (k = 2) estimated based on the validation study of Verleysen et al. [59] for RTM NM-100 and NM-103. 

For the pearlescent pigments, the high degree of aggregation and the presence of mica may result in an underestimation of the real measurement uncertainties. All (100%) of the measured constituent particles were smaller than 100 nm.

The variation among anatase materials and rutile material E171-F was larger than the expanded measurement uncertainties (k = 2) obtained from RTM NM-100 (e.g., for Fmin 79 ± 7 nm to 149 ± 13 nm). Since these materials have a similar size, shape, and agglomeration state as RTM NM-100, the expanded measurement uncertainty is expected to be a realistic estimate. 

Using the optimized sample preparation protocols and image analysis settings, 12 of the 15 materials show a median minimal external dimension, which is expressed as median Fmin, below 100 nm. When the expanded measurement uncertainties (Ucx, k = 2) are added to the median Fmin values, 11 materials have a median minimal external dimension which is significantly smaller than 100 nm (median Fmin + Ucx (k = 2) < 100 nm) (Figure 5). In the E171 materials from the business operators (E171A-E), 18% to 70% of the measured constituent particles were smaller than 100 nm. In the anatase E171 materials purchased at webshops, 64% to 73% of the measured constituent particles were smaller than 100 nm.

To evaluate the possibility of screening E171 materials without prior knowledge on crystallographic composition or the presence of mica, the anatase materials and rutile material E171-F were prepared by protocol P1, as well as by protocol P6 (Table 6). In most cases, P6 resulted in slightly smaller median particle size measurements than P1, which were within the measurement uncertainty budget. 

### 3.5. spICP-MS

The median ESD of the anatase E171 materials and rutile material E171-F, as determined by spICP-MS, ranged from 83 to 125 nm (Table 7). 

The size quantification limit was sample dependent and ranged from 46 to 67 nm. The particle mass concentration varied between 0.68 and 0.87 kg/kg, while the particle number concentration ranged from 0.57 to 1.80 × 10^17^ particles/kg. The median ESD values determined by spICP-MS correspond well with the median values of the Fmin. Nine materials have a median ESD value below 100 nm. When the expanded measurement uncertainties (Ucx, k = 2) are added to the median ESD values, one material has an ESD that is significantly smaller than 100 nm (median ESD + Ucx (k=2) < 100 nm) (Figure 5). No spICP-MS analyses were performed on the pearlescent pigments, as they were not stable in the aqueous dispersions.

## 4. Discussion

An approach was developed and optimized to precisely and accurately characterize the particles in the food additive E171 based on standardized and validated TEM and spICP-MS characterization methods. 

TEM imaging combined with image analysis allowed measuring the particle size and shape distributions, agglomeration state, crystallographic structure, and presence of other compounds and impurities of 15 E171 materials.

Evaluation of selected sample preparation protocols allowed identifying and optimizing the critical factors that determine the measurement of the particle size distribution of E171 materials by TEM. These factors included the pH, the grid charge, the agglomeration state, and the centrifugation time. Controlling these critical factors resulted in an easier particle detection and measurement. In turn, this ensured representative sampling and avoided biased measurements due to agglomeration and particle overlap. It resulted in higher fractions of nanosized particles (<100 nm) than those reported in several publications [6,7,9,42,43,53,56,57], where these factors were less well controlled. Geiss et al. consider such optimization as essential for the development of validated and harmonized sample preparation protocols [55]. Their and our results show that even in relatively simple (food) matrices, the extraction process of particles has an impact on the particle size distribution, underlining the importance of well-planned sample preparation procedures.

In the first instance, representative sampling was established by determining the pH range in which the particles were stable in dispersion based on their zeta potential. Sample preparation at a pH where a strong negative zeta potential was measured allowed obtaining a stable dispersion of TiO_2_ particles, as demonstrated earlier by Guiot and Spalla [77], and representative and uniform coating of the EM grids with particles. Particularly with positively charged Alcian blue-coated grids, artefacts caused by agglomeration were minimized. 

For the examined anatase E171 materials, containing a fraction of (nano)particles, the measured IEP between pH 3 and pH 4, and the negative zeta potential of about −45mV at pH 7, are in agreement with previous studies of food grade anatase TiO_2_ materials [57,78,79]. This IEP of E171 lies below the classical value for bulk anatase [80]. Comparing the zeta potential curves of the E171 materials was shown to permit differentiating anatase E171 materials from pearlescent pigments. This allows the relatively simple screening of pristine E171 (nano)forms before more advanced analyses are performed. This increased zeta potential of the pearlescent pigments is expected to depend on the pH and ionic strength of the dispersion medium [79], the presence of mica layers coated with TiO_2_ particles, the particle size [81,82], and the crystallographic phase [83]. In products containing E171 materials, the above described differentiation becomes unreliable, because the zeta potential will be determined by matrix interferences as well.

The agglomeration state of the material was shown to have an impact on the measurement of the constituent particle size distribution. De-agglomeration of the material was shown to be important for the accurate measurement of constituent particle size. When agglomeration was too high for the anatase materials, image analysis using ellipse fitting often resulted in poor constituent particle identification and in the measurement of multiplet constituent particles as singlets. Due to sub-optimal identification by ellipse fitting, sample preparation protocols based on centrifugation only, with sub-optimal de-agglomeration (P2 and P3), lead to higher median agglomerate and constituent particle sizes than sample preparation protocols with better de-agglomeration by probe sonication (P1, P4, P5, P6).

For pearlescent pigments containing large aggregates of small constituent TiO_2_ particles, automated identification of the constituent particles is difficult. The applied irregular watershed-based protocol [67] results in mean and median values comparable to manual measurements [55] by compensation of over and underestimation of constituent particle size. This can be overcome by improved particle separation (e.g., by intense probe sonication), by the (manual) deletion of wrongly detected particles, and by the application of more advanced segmentation protocols, which can be based on artificial intelligence (AI) [84]. 

Calculating centrifugation times by Stoke’s law ensured that the particle distribution on the grid is representative for the material. Geiss et al confirmed the completeness of the particles’ sedimentation during the centrifugation step by analyzing the supernatant for the presence of particles [55].

For anatase E171 materials and rutile material E171-F, the results obtained from sonication-based protocol P1 and from sonication and centrifugation-based protocol P6 are comparable. However, protocol P6 is more robust, assuring a more reliable sampling and a higher degree of control. Furthermore, for the characterization of E171 particles in a matrix, centrifugation is often required for matrix removal. Therefore, a sonication and centrifugation-based protocol, such as P6, can form the basis of a general standardized protocol for the preparation of E171 samples in a control setting.

The variation amongst the E171 materials was demonstrated by combining validated protocols for TEM specimen (grid) preparation, imaging, and image analysis [59] with an optimized sample preparation protocol. 

The variation measured among the 11 anatase materials reflects the variation in constituent particle size (distribution) of commercial brands of E171 with different undertones. Rayleigh scattering predicts a smaller constituent particle size to give a bluer (“colder”) color, whereas a larger constituent particle size is expected to give a yellower (“warmer”) color [85,86]. The anatase E171 materials obtained from the business operators (E171A–E) better represent the variation of E171 on the market (18% to 70% of constituent particles smaller than 100 nm) than the anatase E171 materials purchased at webshops (64% to 73% of constituent particles smaller than 100 nm). Taking into account the expanded measurement uncertainties (Ucx, k = 2) [59], significantly different median constituent particle sizes are observed among the business operator’s selection. The number-based size and shape distributions of the rutile E171 material (E171-F) was similar to that of the largest anatase E171 material (E171-D).

Geiss et al. show that electron microscopy is currently the only analytical technique that can reasonably be expected to give a quantified measure of the constituent particle size distribution over the full size range for pristine E171 and E171 in products [55]. 

For the anatase materials, the validated spICP-MS characterization methodology that was applied in this study succeeded in obtaining size distributions similar to the TEM-based size distributions: the error bars on the measurements obtained by both techniques overlap for every material. Optimal de-agglomeration, quantitative information of the particle shape, and calculation of the transport efficiency are critical factors to measure the constituent particle size of E171 materials by spICP-MS accurately. Furthermore, quantitative information on the fraction of particles that is smaller than the spICP-MS quantification limit may improve the reliability of the size distribution as well [75]. Corrections for particle shape and missing particle fractions require *a priori* input of quantitative EM analysis. For the examined anatase materials and rutile E171-F, this kind of information was not taken into account in the median ESD calculations, even though the size of the smallest particles observed by TEM is in the order of magnitude of the limit of detection of spICP-MS, hence below the quantification limit. 

For the pearlescent pigments, only EM can give a quantified measure of the constituent particle size distribution: complete de-aggregation is problematic, and the constituent particle size lies below the limit of detection of the spICP-MS methodology. STEM-EDX analysis clearly demonstrated that E171-01, E171-05, and E171-08 are pearlescent pigments of Type I, as defined by JECFA [3]. Commission Regulation (EU) No. 231/2012 stipulates that certain rutile grades of titanium dioxide are produced using potassium aluminum silicate (also known as mica) as a template to form a basic platelet structure, but they requires that all mica is removed during an extractive dissolution process and that the resulting product is a platelet form of rutile titanium dioxide [2]. In all observed cases, a mica layer remained present and was coated with TiO_2_. 

The applied methodology can contribute to the implementation of the EFSA guidance on risk assessment of the application of nanoscience and nanotechnologies in the food and feed chain [87], which states that all dossiers related to nanomaterials have to be accompanied by detailed information on the particle size distribution and on other parameters of the material obtained through validated methods based on suitable analytical techniques. It can also be applied to characterize food and food additives containing a fraction of nanomaterials. Provided that matrix interferences can be avoided in the sample preparation procedure, the proposed approach can be efficiently applied for characterization of E171 in a food matrix. 

## 5. Conclusions

TEM and spICP-MS-based methods were standardized and validated for the physicochemical characterization of E171. A combination of optimized pH, sonication, and centrifugation conditions for TEM sample preparation resulted in the most precise and robust size and shape measurements of constituent particles.

Our results demonstrate significant variation in the particle size and shape distributions, in the crystallographic structure (rutile versus anatase), and in the physicochemical form (pearlescent pigments versus anatase and rutile E171) among representative samples of pristine E171 materials. These factors have to be considered in a risk assessment. 

All the examined E171 materials contain an important fraction of nanoparticles. TEM analysis identified 12 of the 15 E171 materials as being a nanomaterial according to the EC-recommended definition [54], showing a median minimal external dimension (assessed as median Fmin) below 100 nm.

## Figures and Tables

**Table 1:** Specifications and TEM imaging and image analysis conditions for the E171 materials. LLOD: lower limit of detection, LLOQ: lower limit of quantification, ULOD: upper limit of detection, ULOQ: upper limit of quantification.

Reference	Country of Webshop	Magnification	LLOD (nm)	LLOQ (nm)	ULOD (nm)	ULOQ (nm)	Image Analysis Mode
E171-01	France	13,000×	0.83	8.3	3386	338.6	Watershed
E171-02	UK	9300×	1.15	11.5	4730	473.0	Ellipse fitting
E171-03	The Netherlands	9300×	1.15	11.5	4730	473.0	Ellipse fitting
E171-04	UK	9300×	1.15	11.5	4730	473.0	Ellipse fitting
E171-05	The Netherlands	13,000×	0.83	8.3	3386	338.6	Watershed
E171-06	France	9300×	1.15	11.5	4730	473.0	Ellipse fitting
E171-07	France	9300×	1.15	11.5	4730	473.0	Ellipse fitting
E171-08	The Netherlands	13,000×	0.83	8.3	3386	338.6	Watershed
E171-09	France	9300×	1.15	11.5	4730	473.0	Ellipse fitting
E171-A	Not from webshop	9300×	1.15	11.5	4730	473.0	Ellipse fitting
E171-B	Not from webshop	9300×	1.15	11.5	4730	473.0	Ellipse fitting
E171-C	Not from webshop	9300×	1.15	11.5	4730	473.0	Ellipse fitting
E171-D	Not from webshop	9300×	1.15	11.5	4730	473.0	Ellipse fitting
E171-E	Not from webshop	9300×	1.15	11.5	4730	473.0	Ellipse fitting
E171-F	Not from webshop	9300×	1.15	11.5	4730	473.0	Ellipse fitting

**Table 2:** Tested sample preparation protocols for TEM analysis of the E171 materials.

Protocol	P1	P2	P3	P4	P5	P6
Weighed mass	25 mg	88 mg	88 mg	88 mg	88 mg	88 mg
Concentration	2.5 mg/mL	2.5 mg/mL	2.5 mg/mL	2.5 mg/mL	2.5 mg/mL	2.5 mg/mL
pH	10	6–7	6–7	6–7	6–7	6–7
Probe sonication	35 kJ	-	-	10 kJ	10 kJ	10 kJ
Centrifugation	-	30’	2 h	-	30’	2 h

**Table 3:** Applied settings for single particle inductively coupled plasma mass spectrometry (spICP-MS) measurements with inductively coupled plasma mass spectrometry (ICP-MS)/MS.

Instrument Parameter	Operation Settings	
Nebulizer	Micromist	
Spray chamber	Quartz, double pass	
Sampler and skimmer cones	Nickel	
Radio Frequency (RF) power (W)	1550	
Plasma gas flow (L min^−1^)	15	
Auxiliary gas flow (L min^−1^)	0.90	
Carrier gas flow (L min^−1^)	1.04	
Cell gas	10% NH_3_/90% He	
Cell gas flow rate (mL min^−1^)	2	
Sample flow rate (mL min^−1^)	0.47 ± 0.02	
Sampling depth	Ti: 4.3 ± 0.4 Au: 6.6 ± 0.3	
Dwell time (ms)	3	
Sampling time (min)	1	
Transport efficiency (%)	4.8 ± 0.9	
	
Monitored element	Ti	Au
Isotope (amu) monitored at Q1–Q2	48–150	197–197
Elemental composition of the target particle	TiO_2_	Au
Density (g cm^−3^)	4.23	19.3
Mass fraction particle/analyte	1.67	1.0
Ionization efficiency (%)	100	100

**Table 4:** Performance characteristics for the spICP-MS analysis of E171. ESD: equivalent spherical diameter.

	ESD	Particle Mass Concentration	Particle Number Concentration
Repeatability within five series of three repeats (relative standard deviation in percent of average; %)	4.9	18	17
Reproducibility between five series (relative standard deviation in percent of average; %)	8.2	23	13
Size detection limit (nm)	39	-	-
Concentration detection limit (ng/L or particles/L) ^1^	-	50	200
Relative recovery compared (%)	107	96	-
			
Uncertainty budget for the mean of three repeats analyzed on a single day			
Repeatability uncertainty (%)	2.8	10	10
Between-day uncertainty (%)	6.5	23	13
Trueness uncertainty (%)	5.1	11	7
Combined measurement uncertainty (%; k = 1)	8.8	27.5	18
Expanded measurement uncertainty (%; k = 2)	18	55	36

^1^ Concentration detection limit expressed in the diluted dispersion.

**Table 5:** Evaluation of sample preparation protocols based on the medians of the Fmin, Fmax, and aspect ratio (AR) distributions for (**a**) constituent particles and (**b**) agglomerates of anatase E171 material E171-06. For the constituent particles, expanded measurement uncertainties (Ucx, k = 2) are estimated based on the validation study of Verleysen et al. [59] for representative test material NM-100.

**(a) Constituent particles**							
**Protocol**	**P1**	**P2**	**P3**	**P4**	**P5**	**P6, rep 1**	**P6, rep 2**
**Fmin (nm)**	89 ± 8	97 ± 8	98 ± 8	90 ± 8	91 ± 8	88 ± 7	85 ± 7
**Fmax (nm)**	105 ± 10	119 ± 11	119 ± 11	106 ± 10	110 ± 10	105 ± 10	100 ± 9
**AR**	1.16 ± 0.04	1.23 ± 0.05	1.21 ± 0.04	1.16 ± 0,04	1.18 ± 0.04	1.16 ± 0.04	1.14 ± 0.04
**(b) Agglomerates**							
**Protocol**	**P1**	**P2**	**P3**	**P4**	**P5**	**P6, rep 1**	**P6, rep 2**
**Fmin (nm)**	102	263	224	110	149	113	96
**Fmax (nm)**	130	371	327	151	225	164	127
**AR**	1.15	1.39	1.35	1.21	1.30	1.24	1.16

**Table 6:** Analysis results of the 15 E171 materials, including structure and median values of the Fmin, Fmax, and AR distributions obtained by quantitative TEM for samples prepared by protocols P1 and P6. The expanded measurement uncertainties (Ucx, k = 2) are estimated based on the validation study of Verleysen et al. [59] for representative test materials NM-100 and NM-103. The % of constituent particles with Fmin <100 nm, obtained by P1 for pearlescent pigments and by P6 for anatase E171 materials and E171-F, are given.

Reference	Structure	TEM P1			TEM P6			% of Constituent Particles with Fmin <100 nm
		Median Fmin (nm)	Median Fmax (nm)	Median AR	Median Fmin (nm)	Median Fmax (nm)	Median AR	
E171-01	Pearlescent Pigment	30 ± 3	44 ± 5	1.30 ± 0.05	/	/	/	100
E171-02	Anatase	87 ± 7	104 ± 9	1.18 ± 0.04	79 ± 7	94 ± 9	1.17 ± 0.04	74
E171-03	Anatase	88 ± 7	104 ± 9	1.16 ± 0.04	89 ± 8	106 ± 10	1.15 ± 0.04	64
E171-04	Anatase	92 ± 8	112 ± 10	1.18 ± 0.04	86 ± 7	102 ± 9	1.15 ± 0.04	67
E171-05	Pearlescent pigment	20 ± 2	28 ± 3	1.27 ± 0.05	/	/	/	100
E171-06	Anatase	89 ± 8	105 ± 10	1.16 ± 0.04	88 ± 7	105 ± 10	1.16 ± 0.04	65
E171-07	Anatase	83 ± 7	97 ± 9	1.18 ± 0.04	79 ± 7	93 ± 8	1.17 ± 0.04	73
E171-08	Pearlescent pigment	17 ± 2	25 ± 3	1.32 ± 0.06	/	/	/	100
E171-09	Anatase	86 ± 7	103 ± 9	1.17 ± 0.04	84 ± 7	99 ± 9	1.16 ± 0.04	71
E171-A	Anatase	118 ± 10	143 ± 13	1.17 ± 0.04	110 ± 9	128 ± 12	1.14 ± 0.04	40
E171-B	Anatase	97 ± 8	120 ± 11	1.20 ± 0.04	83 ± 7	98 ± 9	1.15 ± 0.04	70
E171-C	Anatase	102 ± 9	127 ± 12	1.22 ± 0/05	94 ± 8	113 ± 10	1.18 ± 0.04	56
E171-D	Anatase	132 ± 11	156 ± 14	1.16 ± 0.04	149 ± 13	178 ± 16	1.18 ± 0.04	18
E171-E	Anatase	92 ± 8	113 ± 10	1.19 ± 0.04	86 ± 7	103 ± 9	1.17 ± 0.04	65
E171-F	Rutile	130 ± 12	168 ± 15	1.26 ± 0.05	139 ± 12	182 ± 17	1.28 ± 0.05	20

**Table 7:** Analysis results of the 15 E171 materials, obtained by spICP-MS, including median values of the ESD distributions, particle mass concentration, and particle number concentrations and their respective expanded measurement uncertainties (Ucx, k = 2; Table 4). The % of constituent particles with ESD <100 nm is given.

Reference	Median ESD (nm)	Particle Mass Concentration (kg/kg)	Particle Number Concentration (particles/kg)	% of Constituent Particles with ESD <100 nm
E171-01	/	/	/	
E171-02	90 ± 16	0.81 ± 0.45	1.68 ± 0.60 × 10^17^	59
E171-03	83 ± 15	0.73 ± 0.40	1.71 ± 0.61 × 10^17^	64
E171-04	91 ± 16	0.82 ± 0.45	1.37 ± 0.49 × 10^17^	56
E171-05	/	/	/	
E171-06	93 ± 16	0.87 ± 0.48	1.44 ± 0.52 × 10^17^	54
E171-07	88 ± 15	0.85 ± 0.47	1.57 ± 0.56 × 10^17^	59
E171-08	/	/	/	
E171-09	89 ± 16	0.81 ± 0.45	1.73 ± 0.62 × 10^17^	58
E171-A	93 ± 16	0.68 ± 0.37	1.27 ± 0.45 × 10^17^	54
E171-B	91 ± 16	0.82 ± 0.45	1.80 ± 0.65 × 10^17^	56
E171-C	102 ± 18	0.74 ± 0.41	1.19 ± 0.43 × 10^17^	48
E171-D	125 ± 22	0.71 ± 0.39	0.57 ± 0.21 × 10^17^	33
E171-E	96 ± 17	0.87 ± 0.48	1.33 ± 0.48 × 10^17^	53
E171-F	125 ± 22	0.72 ± 0.39	0.69 ± 0.25 × 10^17^	32

## Supplementary Materials

The following are available online at https://www.mdpi.com/2079-4991/10/3/592/s1, **Figure S1:** EDX analyses of pearlescent pigments, **Table S1:** Modes, 25^th^ percentiles and 75^th^ percentiles of the Fmin, Fmax, and AR distributions for (**a**) constituent particles and (**b**) agglomerates of material E171-06, **Figure S2:** Annotated TEM images of (**a**) an anatase E171 material analyzed by ellipse fitting and (**b**) a pearlescent pigment analyzed by irregular watershed segmentation, **Figure S3:** Number-based distributions (normalized representation based on kernel density estimation) of the 15 E171 materials obtained by TEM and spICP-MS analysis.
