# Supplementary material for: Physicochemical Characterization of the Pristine E171 Food Additive by Standardized and Validated Methods
Source: Nanomaterials (Basel). 2020 Mar 24;10(3):592. doi: 10.3390/nano10030592 (PMC7153509; doi:10.3390/nano10030592)
Supplement: Supplementary file 1 [file nanomaterials-10-00592-s001.pdf]

Supplementary Materials

Physicochemical Characterization of the Pristine E171 Food Additive by Standardized and Validated Methods

Eveline Verleysen [1,*], Nadia Waegeneers [2], Frédéric Brassinne [1], Sandra De Vos [1], Isaac Ojea Jimenez [1], Stella Mathioudaki [1] and Jan Mast [1]

1 Trace elements and nanomaterials, Sciensano, Groeselenbergstraat 99, 1180 Uccle, Belgium; Frederic.Brassinne@sciensano.be (F.B.); Sandra.DeVos@sciensano.be (S.D.V.); isaac.ojea.jimenez@gmail.com (I.O.J.); stella.mathioudaki@sciensano.be (S.M.); jan.mast@sciensano.be (J.M.)

2 Trace elements and nanomaterials, Sciensano, Leuvensesteenweg 17, 3080 Tervuren, Belgium; nadia.waegeneers@sciensano.be

* Correspondence: eveline.verleysen@sciensano.be; Tel.: +32-2-379-0546

Figure S1. EDX analyses of pearlescent pigments

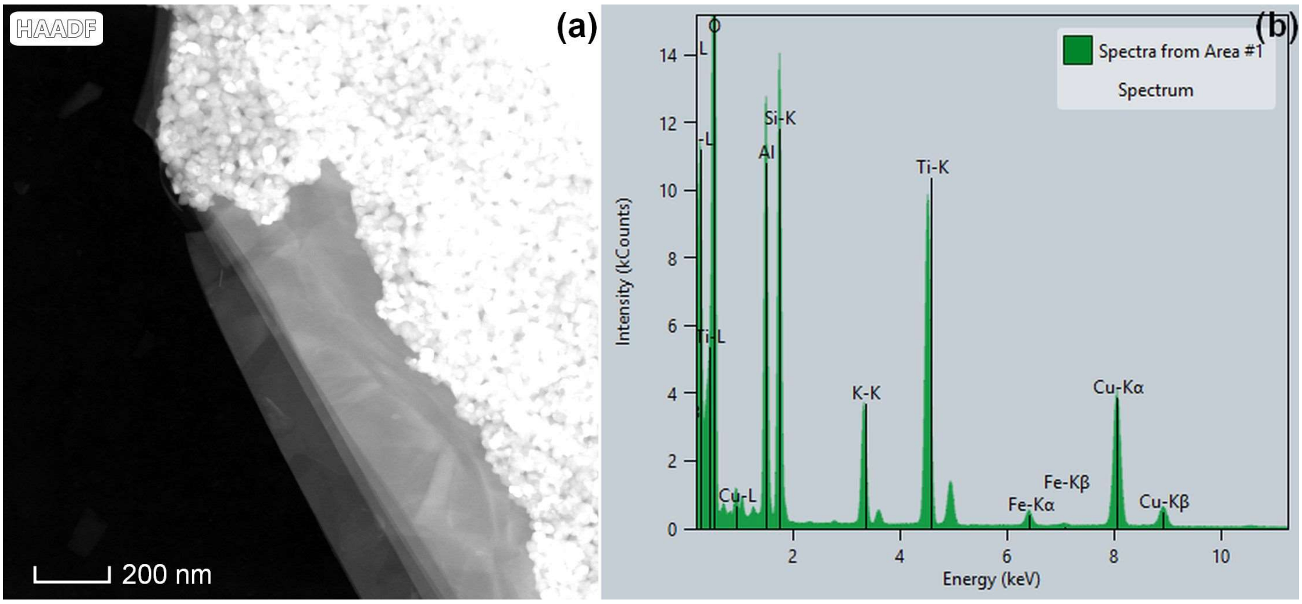

S1A. (a) HAADF-STEM image, and (b) corresponding EDX spectrum of the whole region shown in (a), illustrating the presence of TiO_2 aggregates consisting of many particles on top of mica.

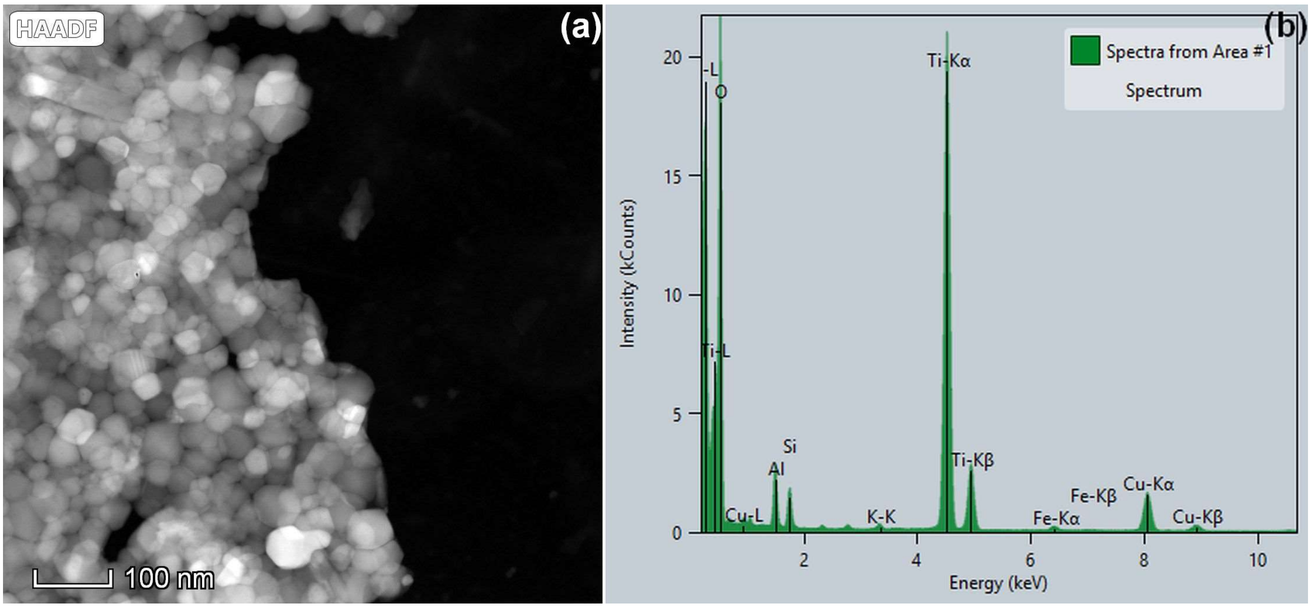

S1B. (a) HAADF-STEM image, and corresponding EDX spectrum of the whole region shown in (a), illustrating the presence of TiO_2 aggregates which contain localized mica parts.

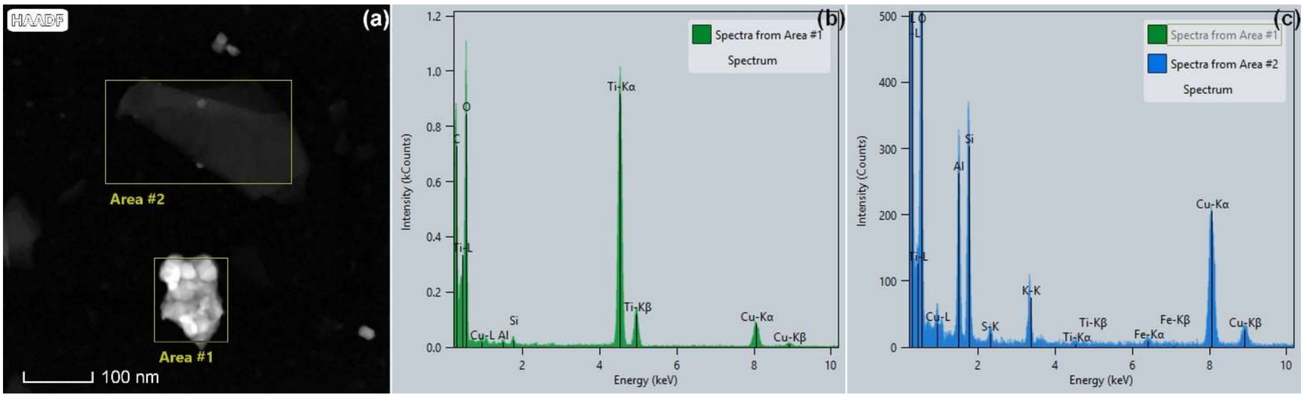

S1C. (a) HAADF-STEM image, and corresponding EDX spectra of area 1 and area 2 indicated on the STEM image shown in (a), illustrating the presence of separated TiO_2 aggregates and mica flakes.

Figure S2. Annotated TEM images of (a) an anatase E171 material analysed by ellipse fitting and (b) a pearlescent pigment analysed by irregular watershed segmentation. Particle measurement is indicated in red.

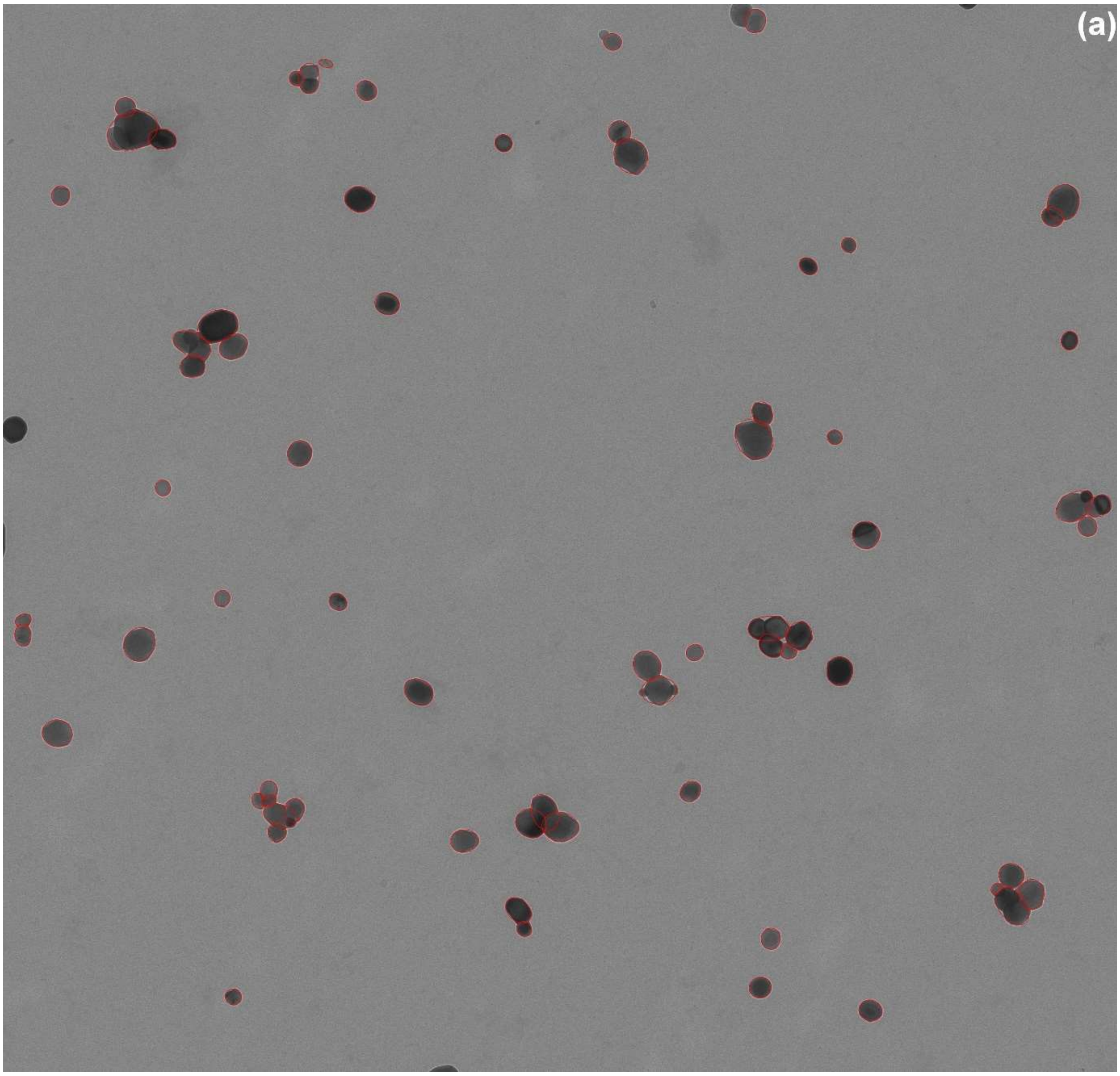

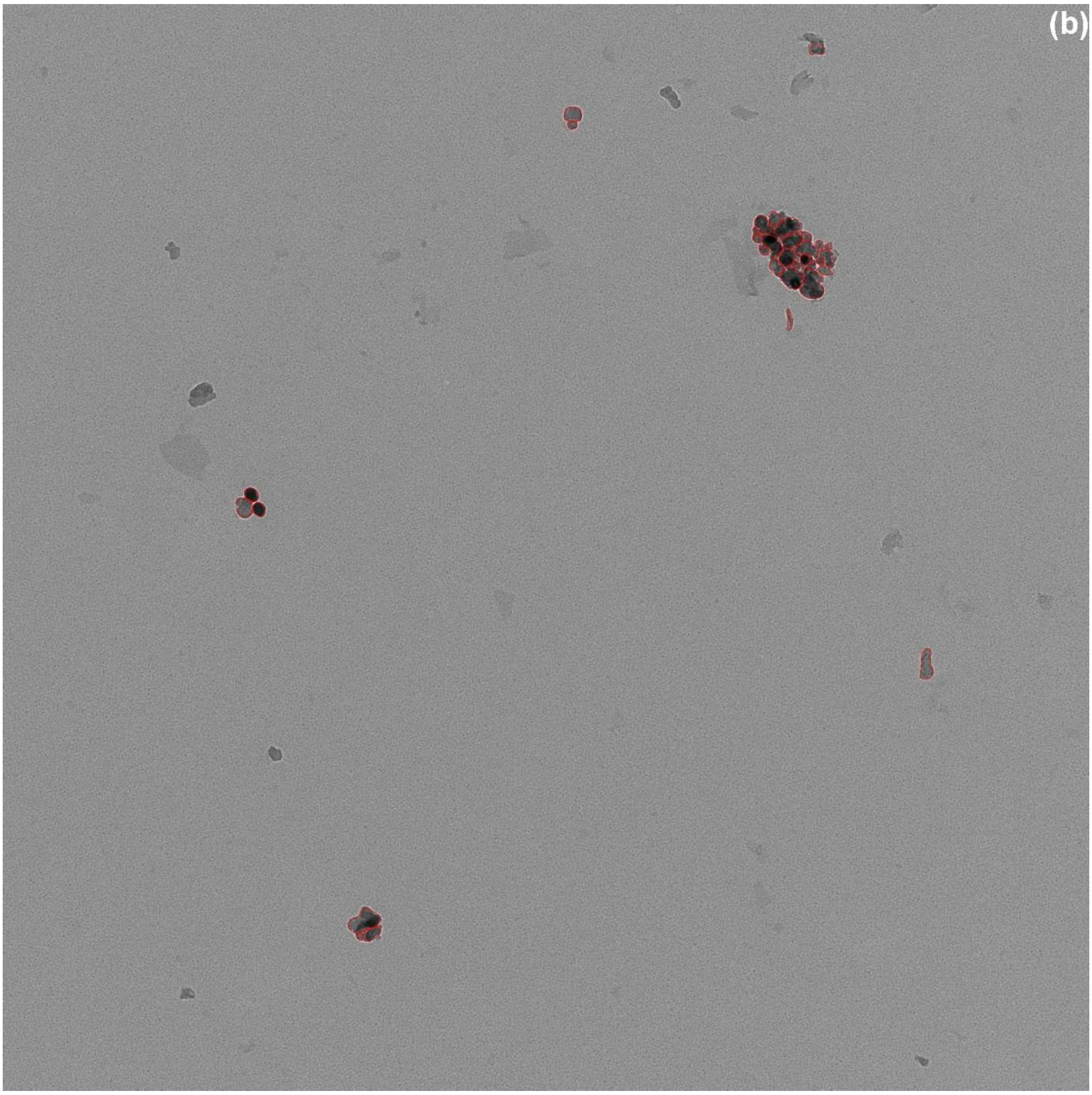
(b)

Figure S3. Number based distributions (normalized representation based on kernel density estimation) of the 15 E171 materials obtained by TEM and spICP-MS analysis. The distributions of the Fmin, Fmax and AR obtained by TEM, prepared by sample preparation protocols P1 and P6 are shown in blue and red, respectively. The ESD distributions obtained by spICP-MS are shown in orange.

E171-01

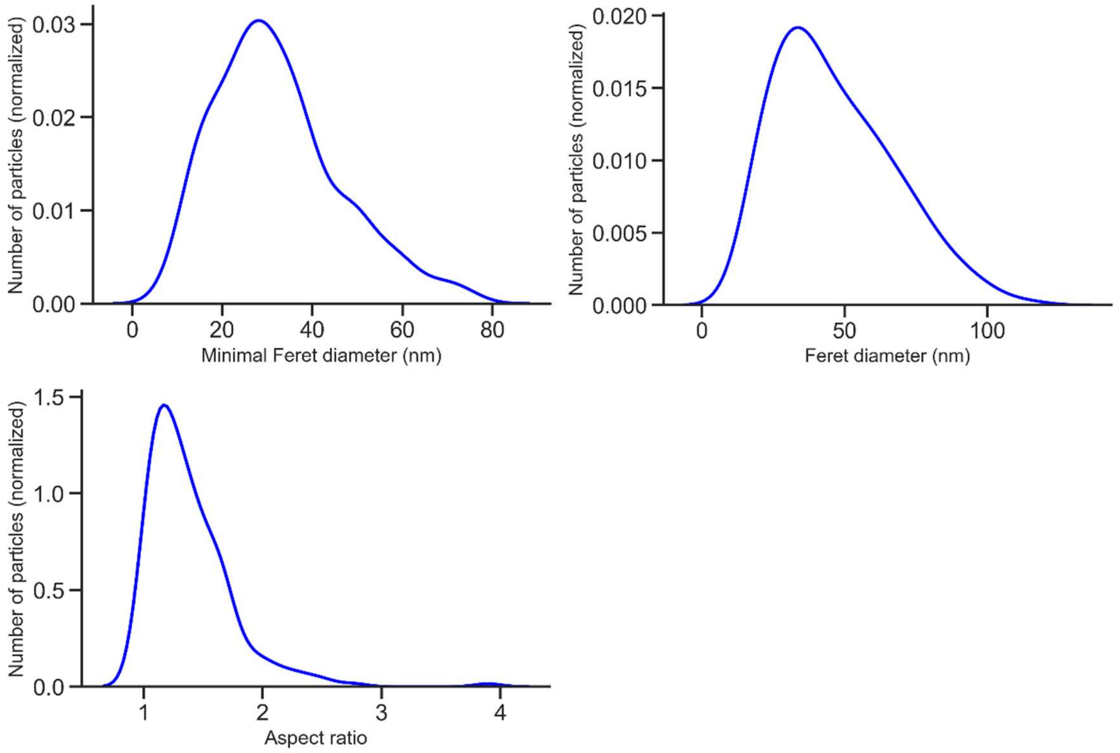

E171-02

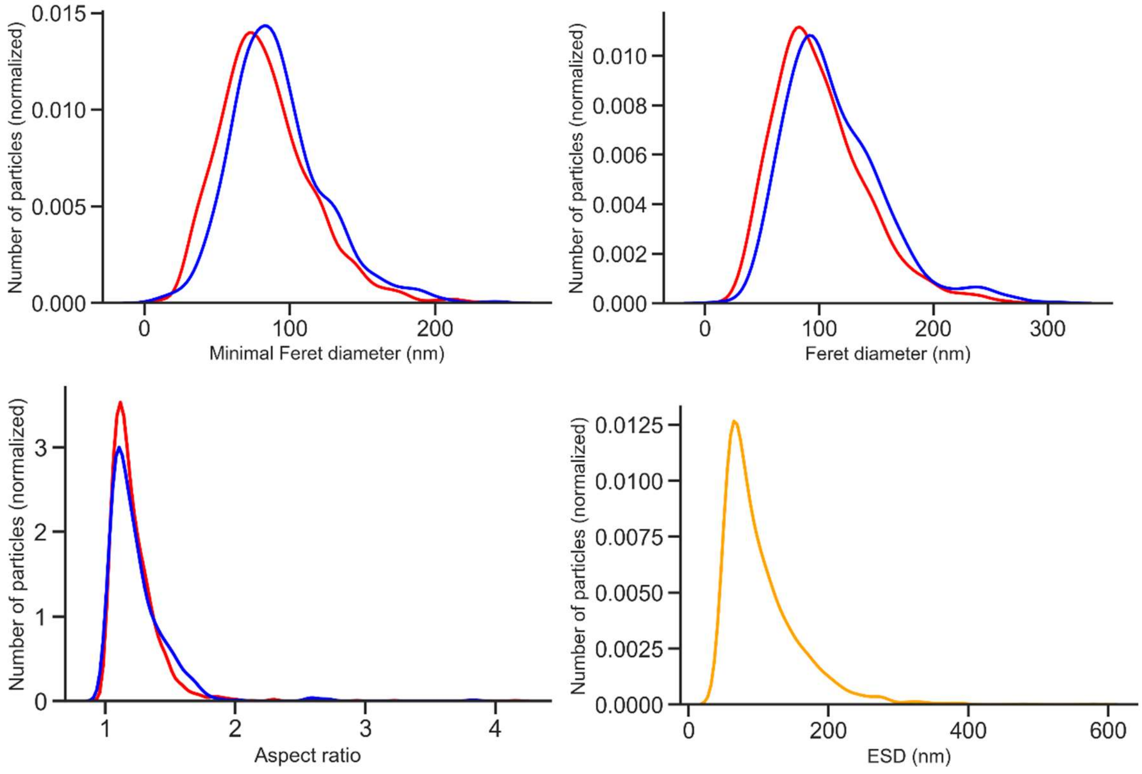

E171-03

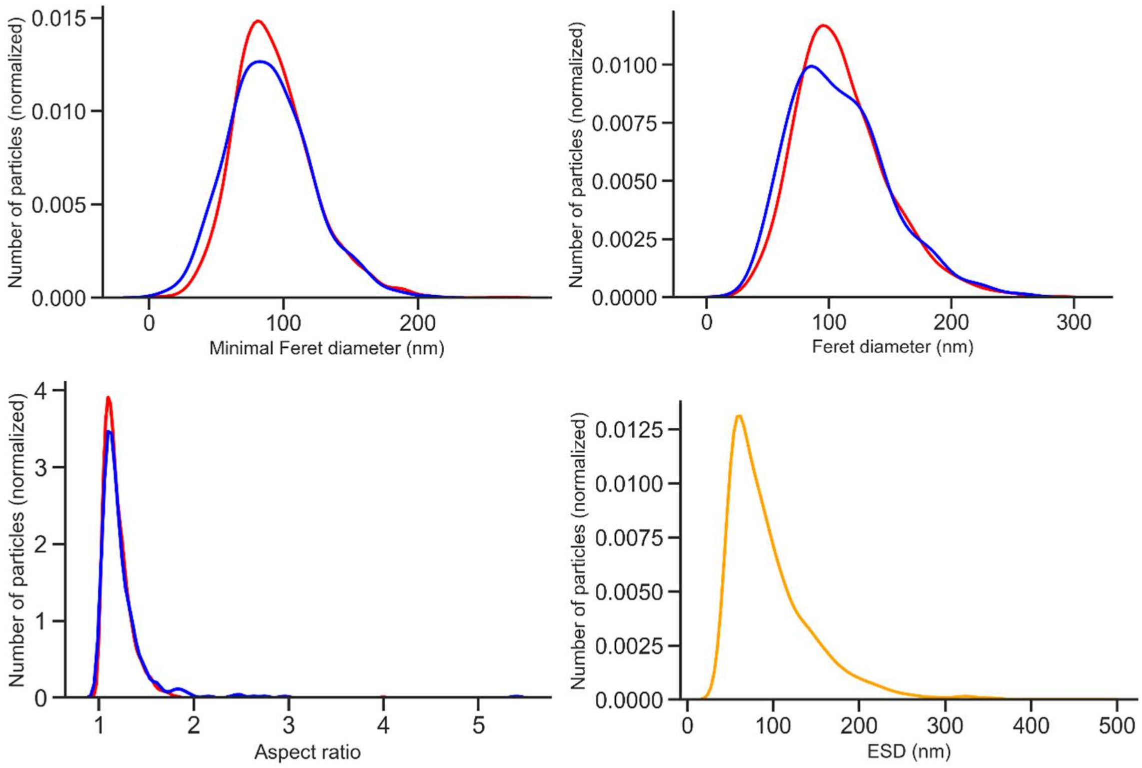

E171-04

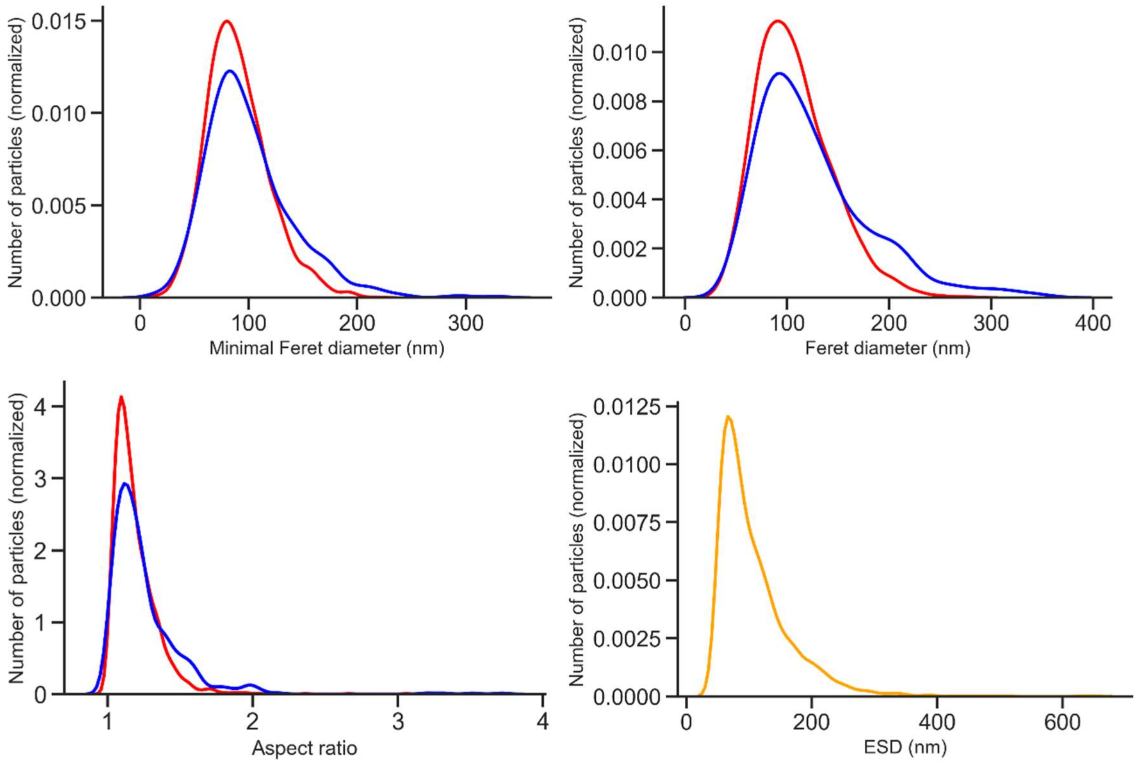

E171-05

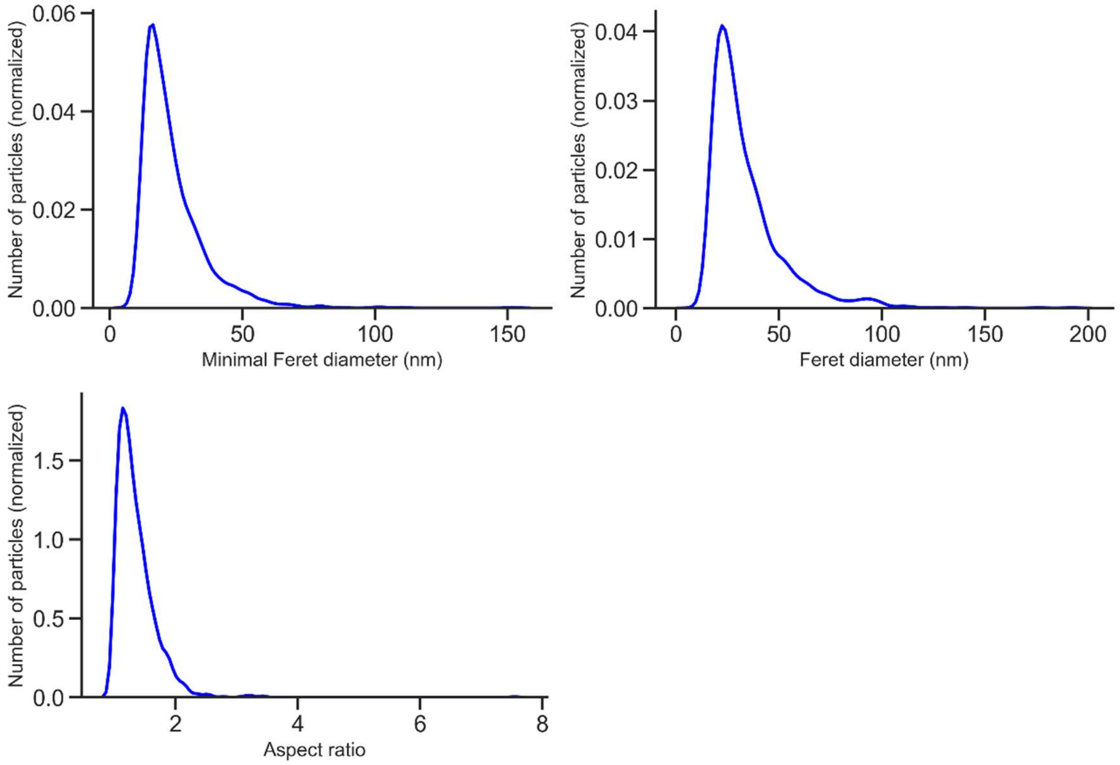

E171-06

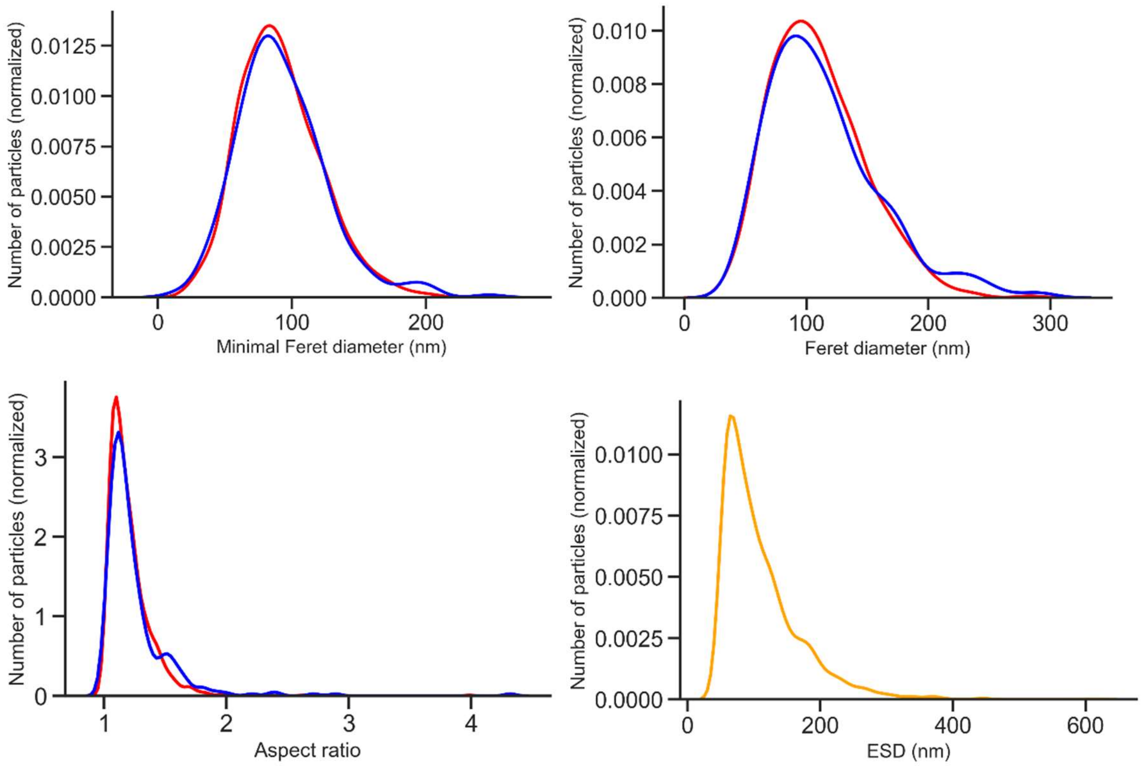

E171-07

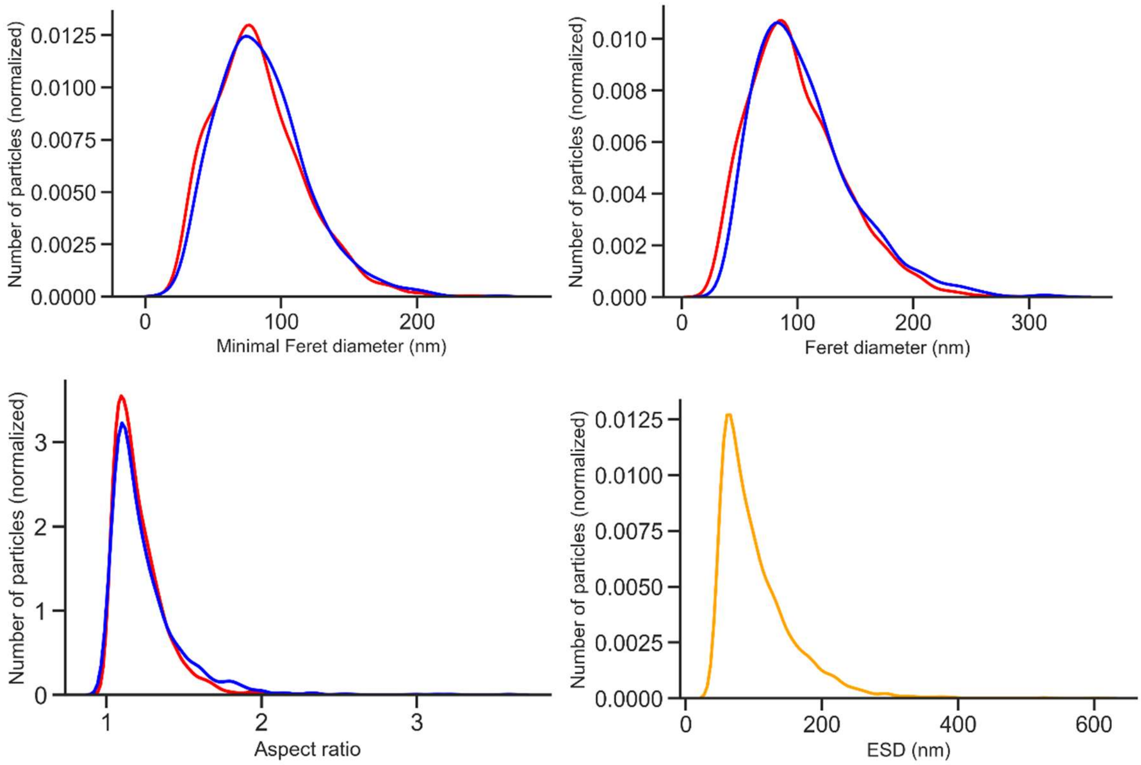

E171-08

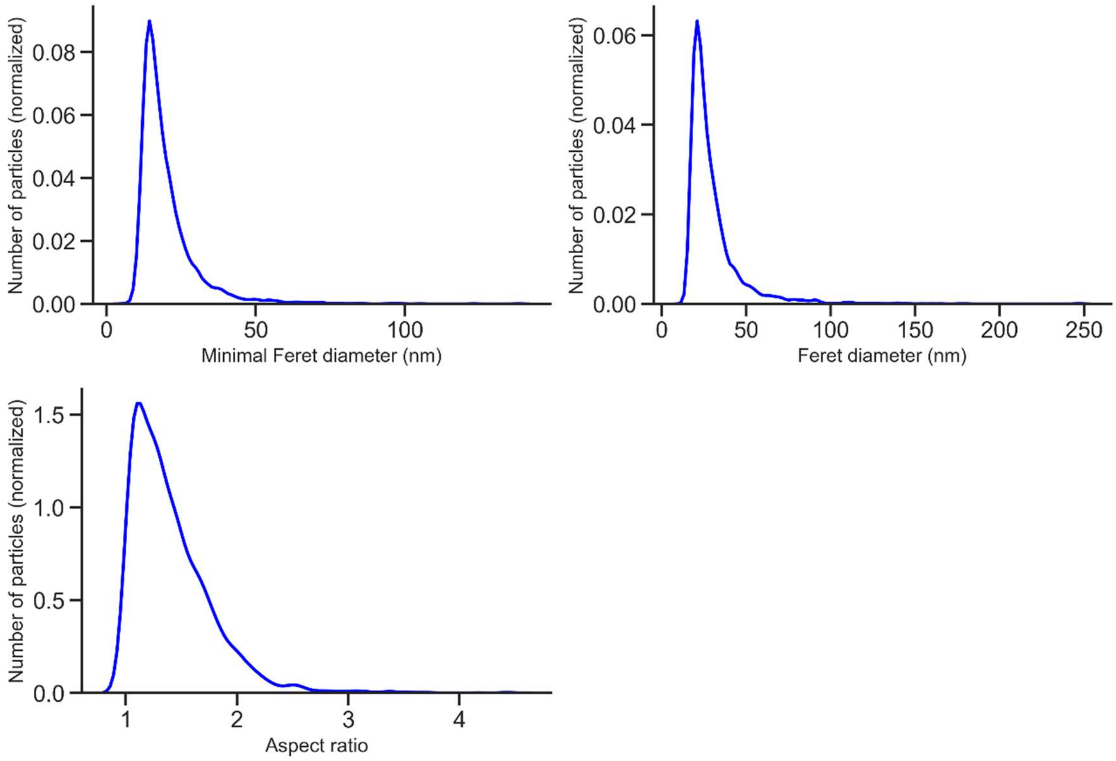

E171-09

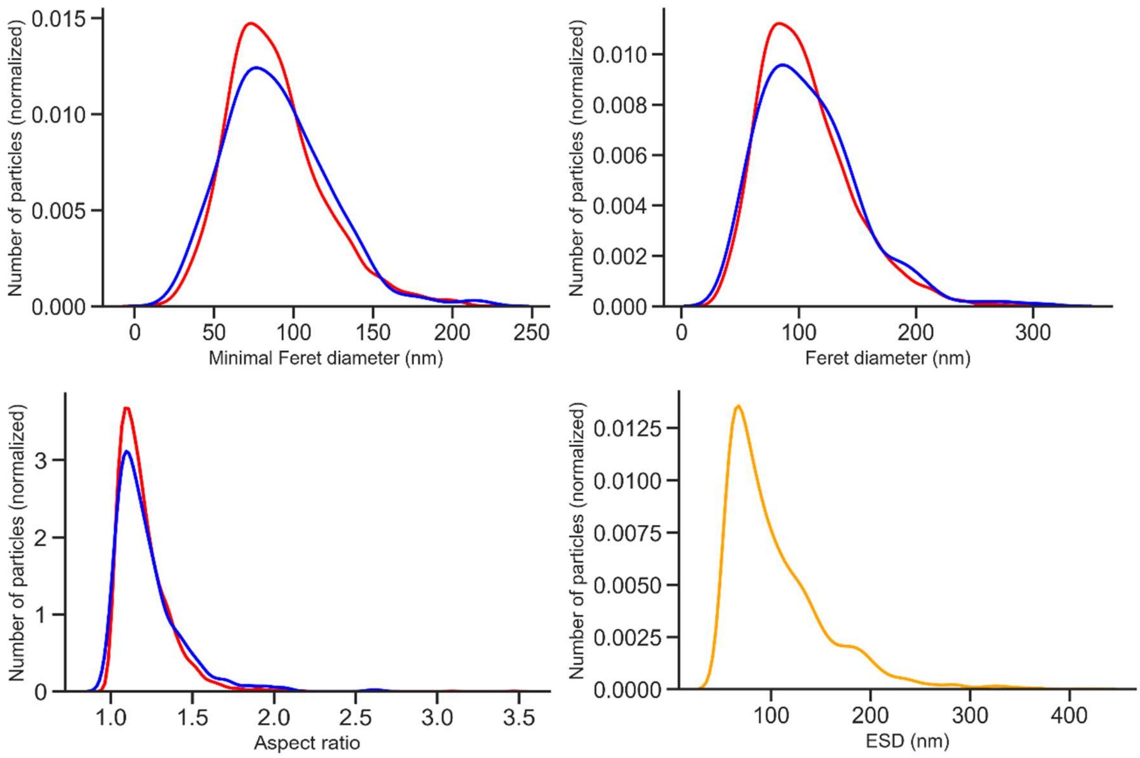

E171-A

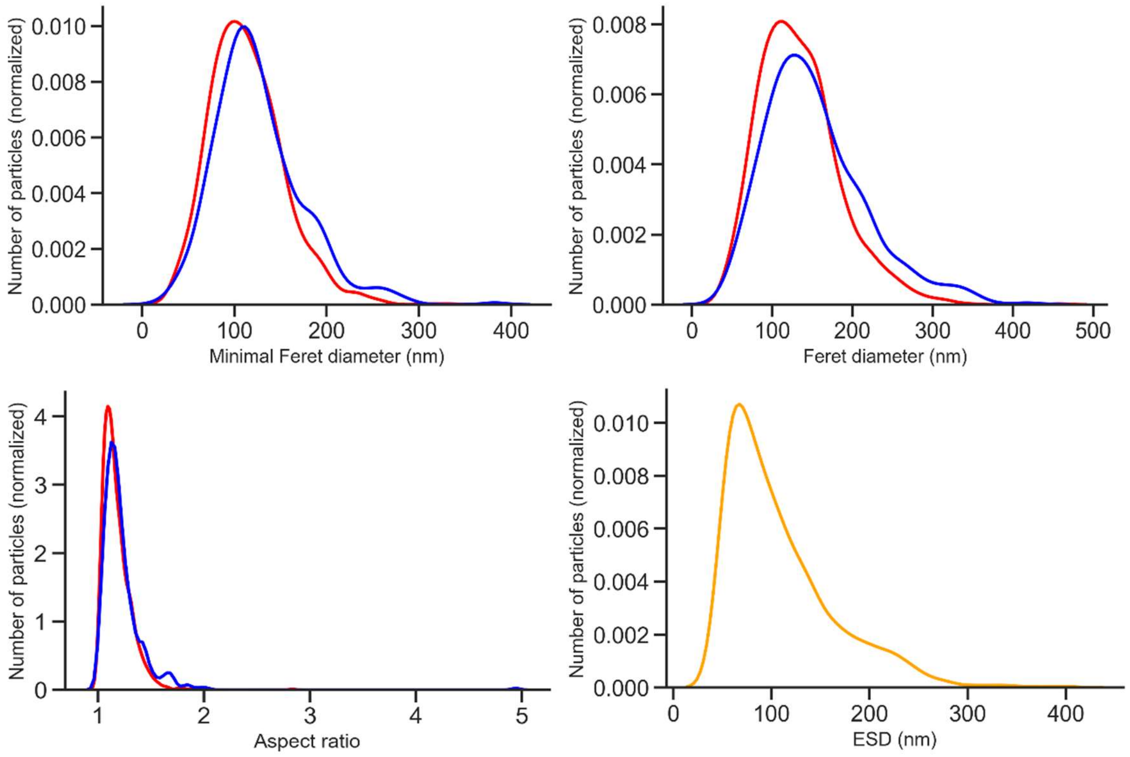

E171-B

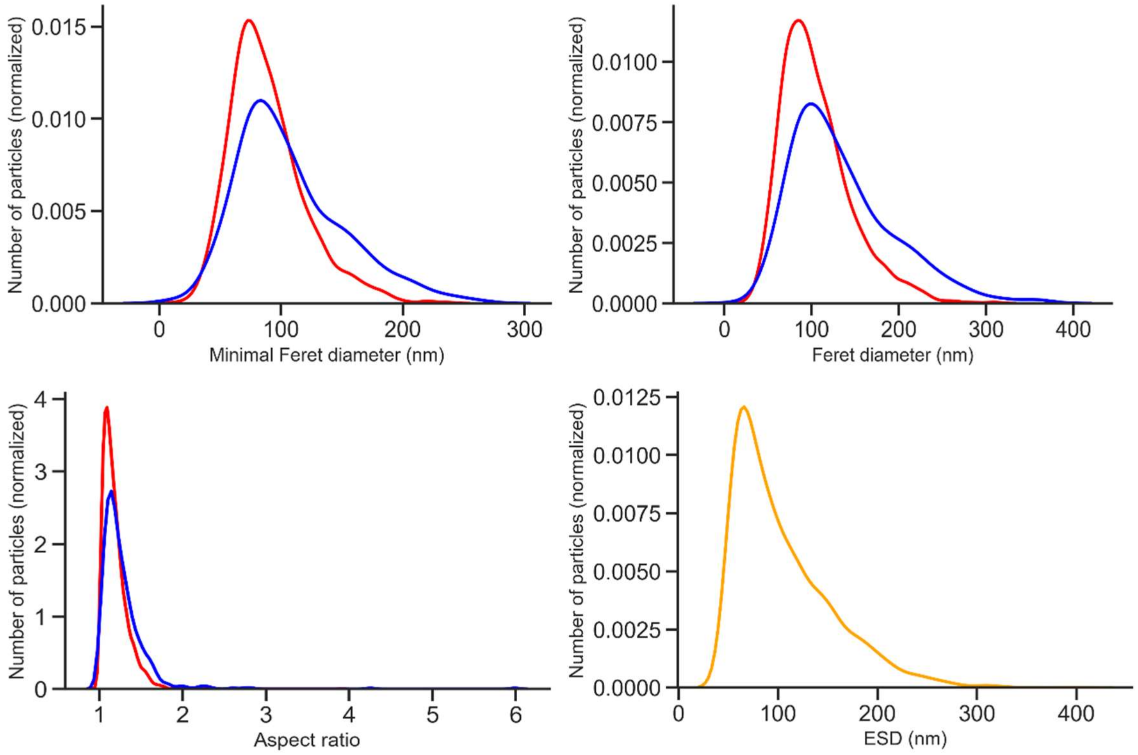

Number of particles (normalized)
Minimal Feret diameter (nm)
Feret diameter (nm)
Aspect ratio
ESD (nm)

E171-C

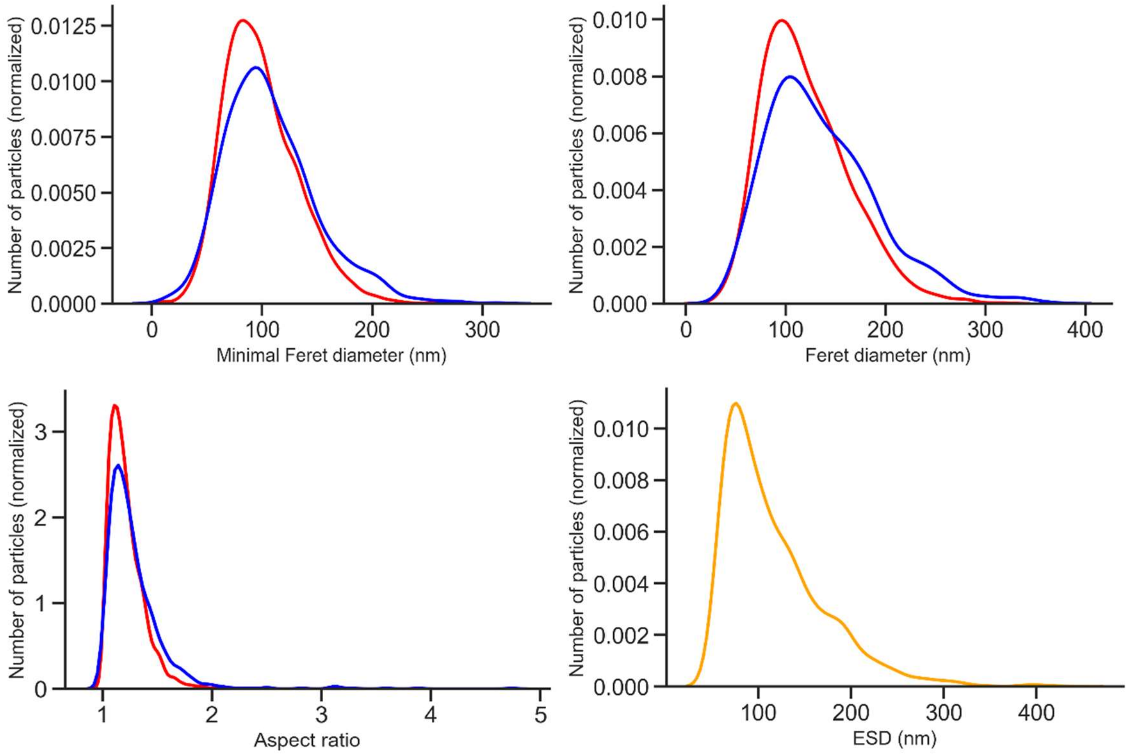

Number of particles (normalized)
Minimal Feret diameter (nm)
Feret diameter (nm)
Aspect ratio
ESD (nm)

E171-D

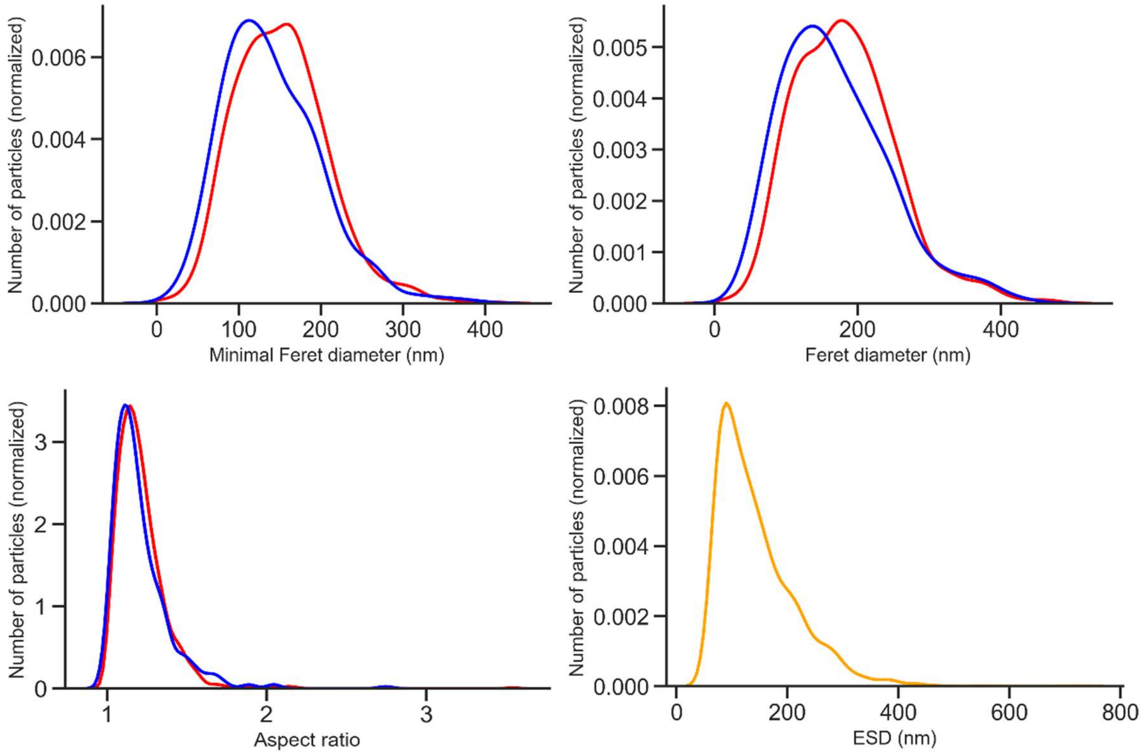
Number of particles (normalized)
0.000
0.002
0.004
0.006
0
100
200
300
400
Minimal Feret diameter (nm)
Number of particles (normalized)
0.000
0.001
0.002
0.003
0.004
0.005
0
200
400
Feret diameter (nm)
Number of particles (normalized)
0
1
2
3
1
2
3
Aspect ratio
Number of particles (normalized)
0.000
0.002
0.004
0.006
0.008
0
200
400
600
800
ESD (nm)

E171-E

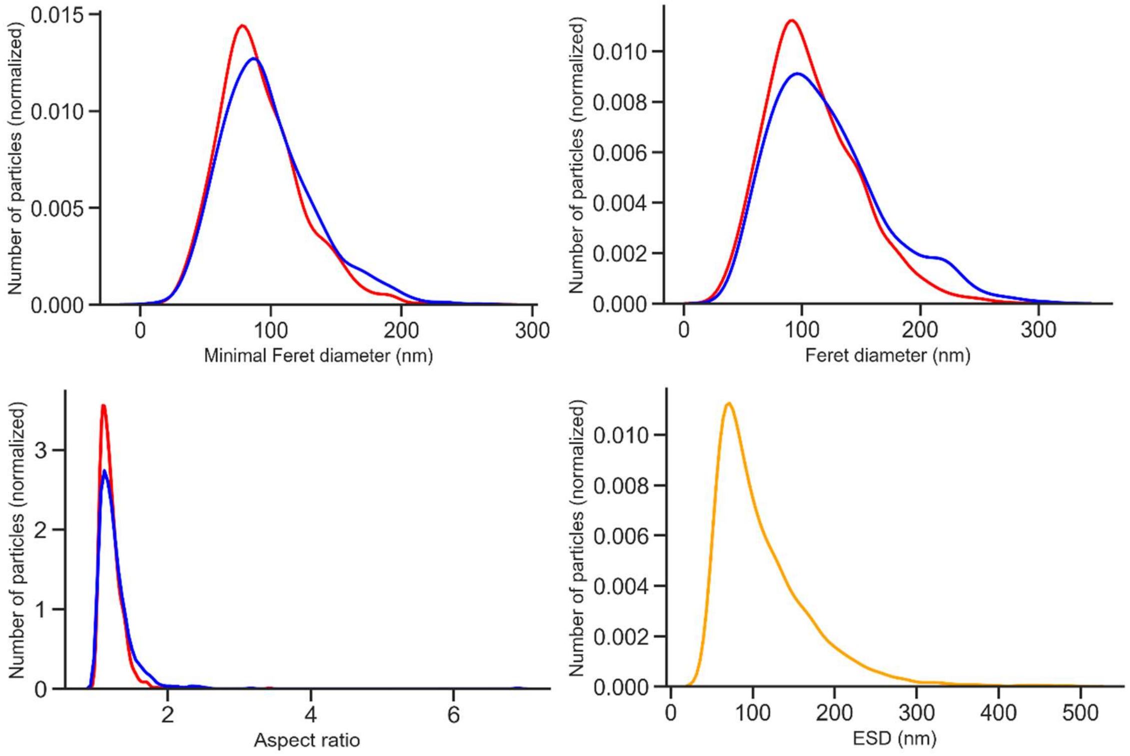
Number of particles (normalized)
0.000
0.005
0.010
0.015
0
100
200
300
Minimal Feret diameter (nm)
Number of particles (normalized)
0.000
0.002
0.004
0.006
0.008
0.010
0
100
200
300
Feret diameter (nm)
Number of particles (normalized)
0
1
2
3
2
4
6
Aspect ratio
Number of particles (normalized)
0.000
0.002
0.004
0.006
0.008
0.010
0
100
200
300
400
500
ESD (nm)

E171-F

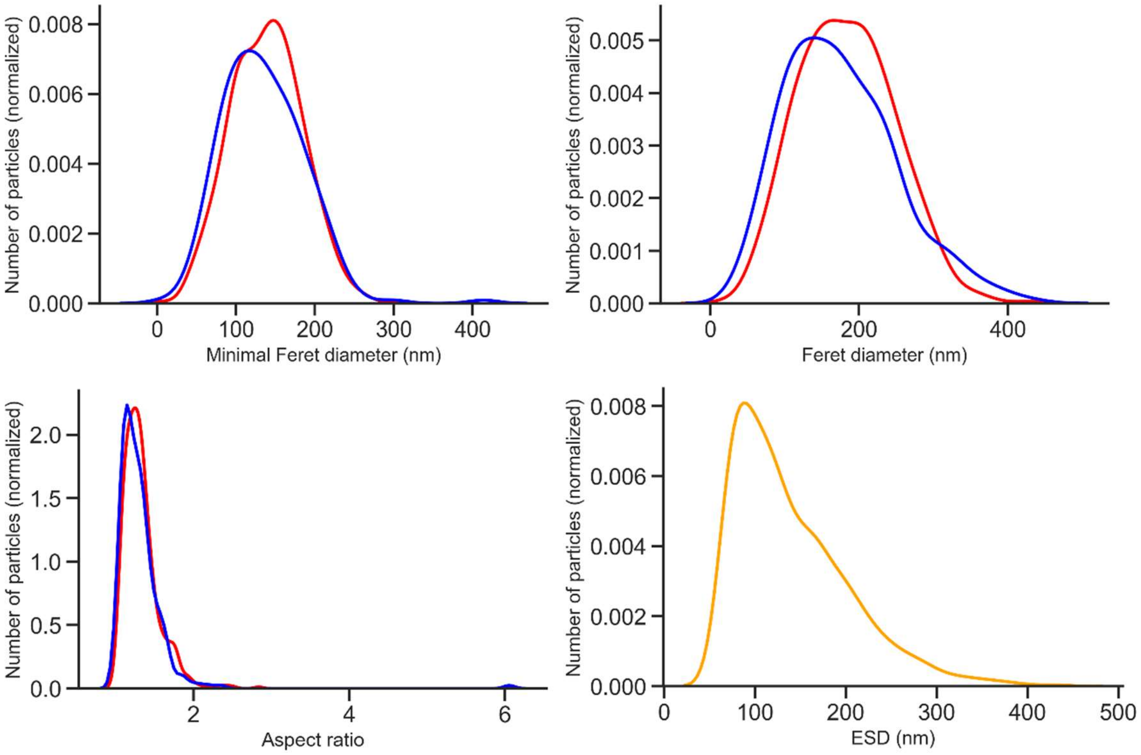

Number of particles (normalized)
0.008
0.006
0.004
0.002
0.000
0
100
200
300
400
Minimal Feret diameter (nm)
0.005
0.004
0.003
0.002
0.001
0.000
0
200
400
Feret diameter (nm)
2.0
1.5
1.0
0.5
0.0
2
4
6
Aspect ratio
0.008
0.006
0.004
0.002
0.000
0
100
200
300
400
500
ESD (nm)

Table S1. Modes, 25 percentiles and 75 percentiles of the Fmin, Fmax and AR distributions for (a) constituent particles and (b) agglomerates of material E171-06.

(a) Constituent particles

Protocol		P1	P2	P3	P4	P5	P6, rep 1	P6, rep 2
Mode	Fmin (nm)	82	85	89	86	82	83	81
	Fmax (nm)	92	108	106	96	97	95	89
	AR	1.135	1.165	1.163	1.098	1.123	1.102	1.090
25%	Fmin (nm)	70	74	75	70	72	70	68
	Fmax (nm)	81	93	92	80	85	81	78
	AR	1.090	1.131	1.122	1.085	1.100	1.086	1.081
75%	Fmin (nm)	111	127	128	113	114	110	106
	Fmax (nm)	137	157	159	136	141	134	128
	AR	1.286	1.362	1.335	1.273	1.309	1.265	1.236

(b) Agglomerates

Protocol		P1	P2	P3	P4	P5	P6, rep 1	P6, rep 2
Mode	Fmin (nm)	86	207	208	92	126	89	81
	Fmax (nm)	102	291	302	93	193	95	89
	AR	1.073	1.310	1.259	1.069	1.156	1.071	1.070
25%	Fmin (nm)	72	162	175	73	101	81	73
	Fmax (nm)	87	219	244	87	138	98	85
	AR	1.040	1.189	1.171	1.053	1.098	1.066	1.051
75%	Fmin (nm)	147	424	300	159	223	160	134
	Fmax (nm)	228	675	454	245	329	235	194
	AR	1.464	1.675	1.641	1.558	1.619	1.533	1.453
